# Unveiling key peak features for olive oil authentication utilizing Raman spectroscopy and chemometrics

**DOI:** 10.1038/s41538-026-00738-2

**Published:** 2026-02-06

**Authors:** Yulong Chen, Renjie Shao, Shan Zeng, Bing Li, Huanjun Hu

**Affiliations:** 1 College of Medicine and Health Science, Wuhan Polytechnic University, Wuhan, China; 2 School of Mathematics and Computer Science, Wuhan Polytechnic University, Wuhan, China

**Keywords:** Chemistry, Mathematics and computing, Optics and photonics

## Abstract

Adulteration of olive oil significantly compromises the interests of both producers and consumers, making its authentication a crucial challenge in the food industry. This study explored the potential of combining Raman spectroscopy with machine learning for discriminating various blended samples and quantifying olive oil content in mixtures. Raman features, such as peak intensities at specific shifts, were extracted from the spectra and analyzed using hierarchical cluster analysis (HCA) and correlation analysis (CA) to identify significant variations corresponding to altered proportions of olive oil. Qualitative and quantitative analyses were performed to classify 10 oil types and predict compositional ratios in binary and ternary blends, comparing different chemometric techniques and input features. Among these, the random forest (RF) model yielded a high classification accuracy (98.9%) and strong predictive performance, with coefficients of determination (*R*^2^) of 0.985 and 0.926 on the binary and ternary samples, respectively. The Shapley additive explanations (SHAP) algorithm was subsequently employed to assess the contribution of key Raman features to the prediction accuracy of superior models. Overall, this novel analytical framework highlights Raman features and offers a promising solution for real-time quality monitoring of olive oil products.

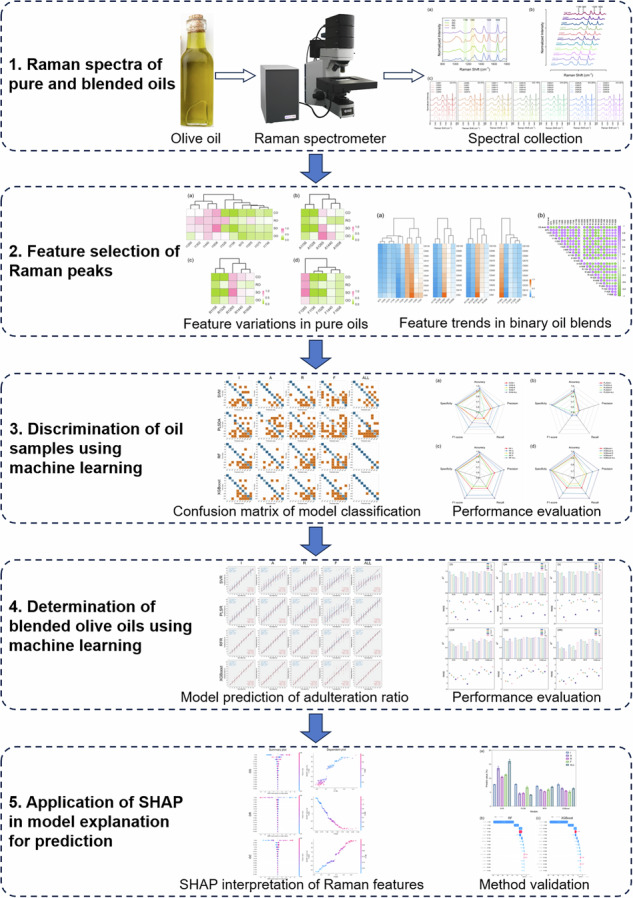

## Introduction

Extra virgin olive oil, a widely traded commodity, is highly sought after for its rich nutritional profile and unique organoleptic attributes^1,2^. Its high market value and complex global supply chain, however, make it a prime target for economically motivated adulteration. High-quality olive oils are often diluted with cheaper alternatives, such as refined olive oils or seed oils. This malpractice not only causes significant economic losses and undermines market fairness but also poses potential health risks to consumers and erodes trust in a product central to the Mediterranean diet^3,4^. Consequently, developing effective methods for detecting olive oil adulteration is not merely a technical challenge but an urgent necessity for ensuring food integrity, protecting public health, and sustaining the economic viability of the olive oil industry.

Traditional laboratory-based methods, such as chromatography coupled with mass spectrometry^5^ and vibrational spectroscopy (e.g., infrared and Raman)^6^, have been commonly employed for olive oil authentication. Among these, Raman spectroscopy, known for its rapid and non-destructive nature, has emerged as an effective tool for detecting adulterated olive oils. For instance, Fan et al. employed confocal Raman spectroscopy in combination with multivariate statistical techniques to quantitatively analyze the components of ternary blended olive oils^7^. Portarena et al. developed a novel method for determining the relative ratios of lutein and β-carotene in olive oil through curve fitting of Raman bands, aiding food chemical assessments^8^. Using a portable spatially offset Raman spectroscopy (SORS) device, Jiménez-Hernández et al. authenticated extra virgin and virgin olive oils against sunflower oil adulteration by applying multivariate regression models^9^. Raman spectroscopy provides rich information due to the distinct molecular vibration modes and changes in polarizability during Raman scattering, which is useful for identifying chemical bonds for structural characterization and even quantifying active ingredients. Furthermore, Hua et al. combined Raman spectra with the peak-area-ratio approach to monitor the quality of flaxseed oil during frying, correlating feature peak areas and ratios with parameters such as acid and peroxide values^10^. Despite the promise of Raman spectroscopy for olive oil authentication, existing approaches primarily rely on single metrics like peak intensities, thereby overlooking the complementary spectral information embedded in multidimensional attributes such as peak area and relative ratios. Integrating these features is crucial for developing more effective and robust models, especially for detecting subtle adulteration under realistic, variable conditions.

Recent advancements in machine learning algorithms for handling complex data have led to significant breakthroughs in food analysis^11^. Soares et al. developed an interval support vector machine (SVM) model using Raman spectroscopy to discriminate certified omega-3 fish oils, achieving 96% accuracy on the internal validation set^12^. Moe Htet et al. applied Raman spectra in combination with the partial least squares (PLS) method to classify vegetable oil samples and measure α-tocopherol contents, demonstrating strong predictive performance^13^. Chen et al. explored several machine learning models combined with Raman signals for the quantitative analysis of β-carotene and unsaturated fatty acids in blended sunflower seed-olive oils, with RF outperforming other models in prediction accuracy^14^. Utilizing surface-enhanced Raman spectroscopy (SERS) data, Xu et al. applied the extreme gradient boosting (XGBoost) model to identify pure and adulterated camellia oils, achieving relatively high accuracy on the test set^15^. However, the “black-box” nature of machine learning models presents challenges in interpreting their predictive results^16^. The SHAP algorithm has become widely used to interpret model decisions by evaluating the importance of input features. For instance, Abdalla et al. integrated Raman scattering with chemometrics to determine drug release from polysaccharide coatings, then employed SHAP analysis to identify key factors influencing predictions, such as time and incubation medium, in the RF model^17^.

In this study, Raman spectroscopy combined with chemometrics was applied to authenticate blended olive oils for content determination. Initially, Raman spectral data from pure and blended oils were collected, and key peak features, including peak intensity (I), peak area (A), peak area ratio (R), and full width at half maximum (F), were analyzed using HCA and heatmap visualization. The CA was then used to explore the relationship between increasing proportions of olive oil and corresponding Raman features in binary blends. To classify oil samples and quantify the olive oil content, machine learning models, including SVM, PLS, RF, and XGBoost, were employed. Model performance was evaluated to identify the most suitable models and relevant Raman features for olive oil authentication. In quantifying the olive oil content in mixtures, the SHAP was applied for feature selection and model interpretation, ranking the significance of Raman features and explaining their impact on content estimation. Notably, the commercially available blended oils were used for method validation, and the results from various models were further explained using SHAP analysis. This study introduces an innovative framework by comparing multiple Raman peak features in machine learning models for the quantification of olive oil in binary and ternary blends, offering a valuable screening tool for detecting adulteration in high-value edible oils.

## Results and discussion

### Raman spectra of pure and blended oils

**Fig. 1:**
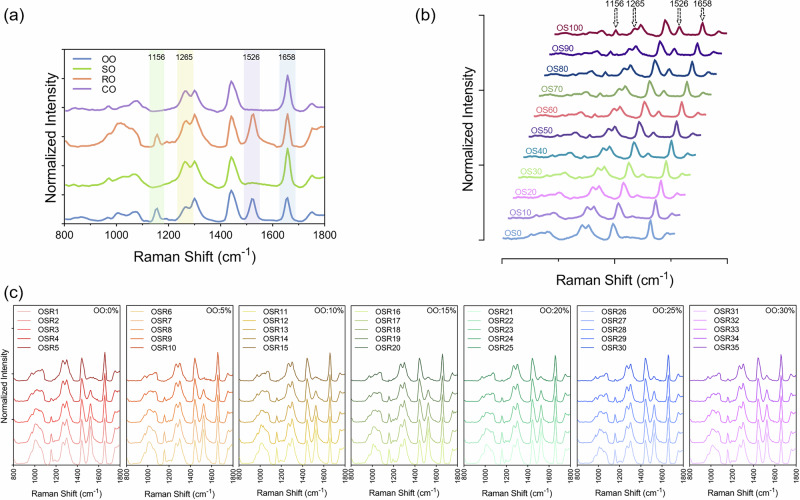
Average Raman spectra of various oil samples. **a** Four pure edible oils: olive oil (OO), sunflower seed oil (SO), rapeseed oil (RO), and corn oil (CO), showing differential Raman feature bands; **b** Binary blended olive-sunflower seed oils (OS) at different mixing proportions; **c** Ternary blended oils (OSR) composed of olive, sunflower seed, and rapeseed oils at varying mixing proportions.

Figure 1a shows the average Raman spectra of four types of pure oils, such as olive oil, after intensity normalization. The main characteristic bands are observed in the range of 800-1800 cm^-1^, corresponding to specific molecular vibration modes. It is evident that four Raman peaks at 1156, 1265, 1526, and 1658 cm^-1^ exhibit distinct signals in the pure oil samples. Their spectral features, such as peak intensity and peak area, were further analyzed to differentiate olive oil from other edible oils. The variations in Raman spectra are mainly due to differences in the molecular composition of the oils, including bioactive compounds like polyphenols, carotenoids, and unsaturated fatty acids. For example, the concentration of β-carotene varies significantly across olive oil varieties, depending on factors like cultivar, geographical origin, and harvesting methods^18^. The Raman peak at 1440 cm^-1^, attributed to the deformation vibration of the –CH₂ group, shows relatively high and stable intensity across all pure oil samples^19,20^; therefore, this Raman peak was used to normalize the entire spectrum. Based on vibrational assignments and functional groups, the Raman peaks at 1156 and 1526 cm^-1^ are characteristic of β-carotene, which is notably present in olive oils^21^. The former corresponds to the C–C stretching vibration of the –(CH_2_)_n_– group, and the latter is associated with the C = C stretching vibration of the RHC = CHR group^14,22^. Additionally, the Raman bands at 1265 and 1658 cm^-1^ correspond to the =C–H symmetric rocking and C = C stretching vibrations, respectively, both of which are related to the cis RHC = CHR group and are closely associated with unsaturated fatty acids^23,24^. Despite slight variations in feature peak intensity, different brands of pure edible oils from different brands and origins show high spectral similarity. Notably, characteristic bands of olive oil are evident around 1156 and 1526 cm^-1^ when compared to other edible oils, which agrees with previous findings^20,21^. However, these two Raman signals also appear in rapeseed oil, which may lead to misclassification and prediction errors in blended samples. The spectral overlap between olive and rapeseed oils at 1156 and 1526 cm^-1^ presents a challenge for accurate classification, highlighting the need for complementary analytical techniques or more advanced algorithms to improve classification accuracy.

**Fig. 2:**
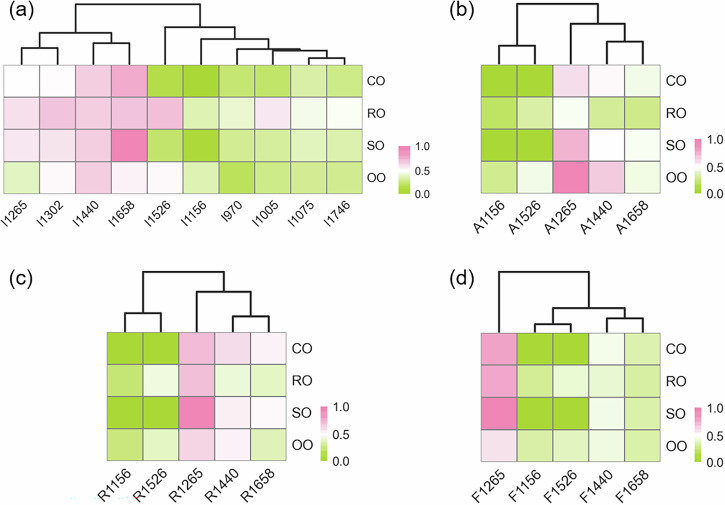
HCA and heatmap visualization of Raman features for various pure oils. **a** Peak intensity (I); **b** Peak area (A); **c** Peak area ratio (R); **d** Full width at half maximum (F).

As displayed in Fig. 1b, the offset spectrogram illustrates the variation in Raman characteristic peaks for representative binary blends of olive-sunflower seed oils (OS) with increasing olive oil content. Specifically, OS0 represents 0% olive oil (pure sunflower seed oil), while OS100 represents 100% olive oil (pure olive oil). As the proportion of olive oil increases in the OS blends, the intensity of Raman bands at 1156 and 1526 cm^-1^ becomes more pronounced, with clearer peak shapes. In contrast, the Raman signals at 1265 and 1658 cm^-1^ gradually decrease from OS0 to OS100. This trend can be explained by previous studies, which show that olive oil is richer in oleic acid, while sunflower seed oil contains higher levels of linoleic acid in the unsaturated fatty acid profile^14,20^. Additionally, the Raman spectra of other binary blends, including olive-rapeseed oils (OR) and olive-corn oils (OC) at various mixing proportions, are shown in S. Fig. 1. Similarly, Fig. 1c presents the Raman spectra of representative ternary blends (OSR) composed of olive, sunflower seed, and rapeseed oils at varying blending ratios. In seven different mixing proportions, ranging from 0% to 30% olive oil, the change in feature peaks is clearly observed, with increasing rapeseed oil content leading to higher Raman intensity at 1156 and 1526 cm^-1^ from OSR1 to OSR5. In contrast, higher olive oil content slightly enhances the signals of these two characteristic bands. The Raman spectra of other ternary blends, including OSC (olive, sunflower seed, and corn oils) and ORC (olive, rapeseed, and corn oils) at different mixing proportions, are shown in S. Fig. 2. The spectral differences observed in both pure oils and blended samples are crucial for olive oil authentication and quality control. Raman spectroscopy effectively differentiates oils based on bioactive compounds such as β-carotene^14^ and unsaturated fatty acids^25^, which contribute to the health benefits and sensory properties of olive oil. This technique offers a valuable tool for detecting adulteration and ensuring the authenticity of premium olive oils, especially in regions susceptible to fraud.

### Feature selection of Raman peaks

Figure 2 shows the HCA and heatmap visualization of various Raman features, including intensity (I), area (A), ratio (R), and full width at half maximum (F), across four classes of pure oils. For practical olive oil identification, the distinct Raman feature peaks are critical for differentiating olive oil from other edible oils. These differences arise due to the unique molecular composition of olive oil, which contains higher concentrations of bioactive compounds such as β-carotene and oleic acid. These compounds contribute to the distinct Raman peaks at 1156 and 1526 cm^-1^, which are not as prominent in other oils. The HCA heatmap of these Raman features highlights key peak attributes that influence classification results. This visualization technique is particularly valuable in complex blends, allowing for the identification of critical features that can differentiate oils with similar chemical compositions. For example, olive oils exhibit relatively higher intensities at I1156 and I1526, and lower intensities at I1265 and I1658 compared to other oils. Regarding peak area and area ratio, olive oil shows higher values for A1156, A1526, A1265, R1156, and R1526. Additionally, low F1265, along with high F1156 and F1526, is observed in olive oil. These differential Raman features are subsequently used as inputs for the qualitative and quantitative analysis of blended olive oils, providing valuable insights into the composition and authenticity of olive oil blends. Such Raman spectral data can be leveraged in quality control and adulteration detection, offering a non-destructive and efficient method for olive oil authentication.

**Fig. 3:**
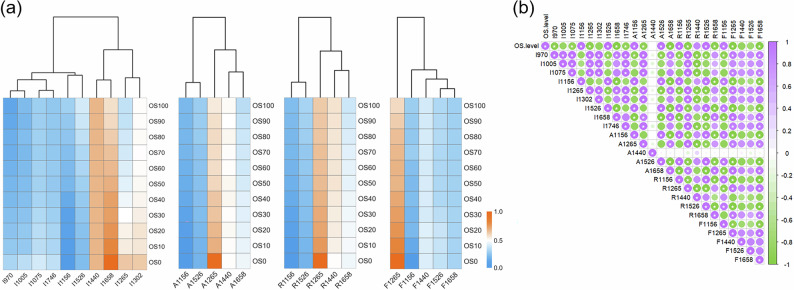
Differences in Raman features of binary blended olive-sunflower seed oils (OS). **a** Hierarchical cluster analysis (HCA); **b** Correlation analysis (CA) between olive oil content levels and Raman features.

Figure 3a presents the HCA and heatmap visualization of various Raman features in binary blends of olive-sunflower seed oils (OS). Increasing olive oil content is positively correlated with higher normalized peak intensities at 1156 and 1526 cm^-1^ (I1156 and I1526). These Raman bands are attributed to the unique presence of β-carotene in olive oil, which is absent in sunflower seed oil^8,14^. The higher β-carotene concentration in olive oil is associated with its potent antioxidant properties^26^, which contribute not only to its health benefits but also to its distinct spectral fingerprint, making it a key parameter for quality and authenticity assessment of olive oil. Additionally, the normalized peak intensities at 1265 and 1658 cm^-1^ gradually decrease as the proportion of olive oil increases. This can be explained by the higher oleic acid content in olive oil and the higher linoleic acid content in sunflower seed oil, as both acids differ in their unsaturated fatty acid profiles^14,20,25^. Linoleic acid, with the same carbon chain length as oleic acid but an additional C = C double bond, produces stronger Raman intensities at 1265 and 1658 cm^-1^ in sunflower seed oil. This explains the observed decrease in I1265 and I1658 in OS samples with higher olive oil content. Notably, the different unsaturated fatty acid profiles in olive and sunflower seed oils could also impact their sensory properties, such as taste and stability, further underscoring the importance of distinguishing between these oils in blended products. Parallel analysis of peak area, peak area ratio, and full width at half maximum at varying Raman shifts further reveals key Raman features for olive oil authentication, which could be crucial for quality control and adulteration detection in the olive oil industry.

**Fig. 4:**
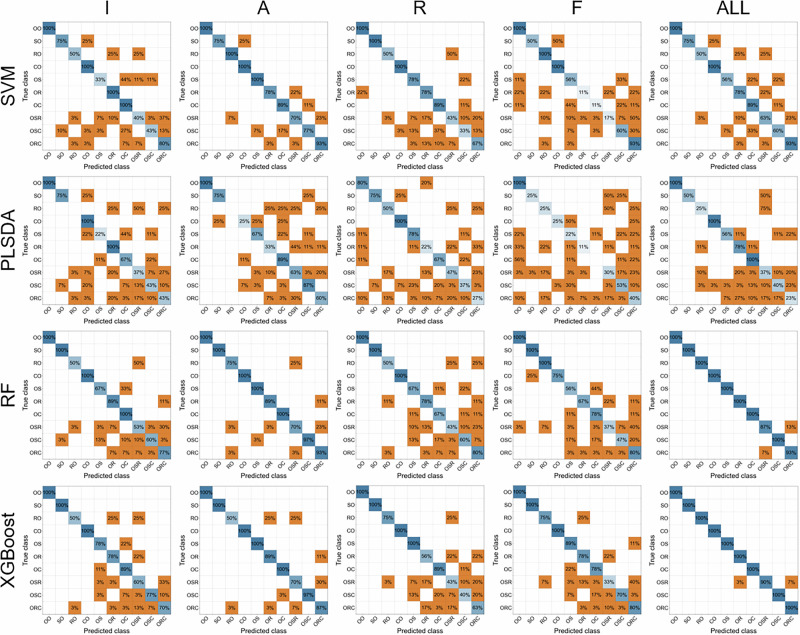
Confusion matrices of model classification based on Raman features of pure and blended oils.

As shown in Fig. 3b, the correlation between olive oil content levels in OS blends and Raman features was analyzed using CA. The results reveal significant positive correlations (*p* < 0.05) between OS levels, ranging from 0% to 100% olive oil content, and I1156, I1526, A1156, A1526, R1156, R1526, and F1156. These correlations are likely due to the higher concentrations of β-carotene and oleic acid in olive oil, which contribute to the distinct Raman peaks at 1156 and 1526 cm^-1^. The positive correlations with A1156 and A1526 further emphasize the role of the peak areas in characterizing the oil composition, as the increase in olive oil content leads to more pronounced Raman bands associated with these bioactive compounds. Conversely, significant negative correlations (*p* < 0.05) were found with I1265, I1658, A1265, A1658, R1265, R1658, F1265, and F1658. This decrease in Raman signals at these wavelengths can be attributed to the higher linoleic acid content in sunflower seed oil, which exhibits different molecular characteristics, such as additional C=C double bonds, contributing to stronger Raman intensities at 1265 and 1658 cm^-1^. In the CA of various Raman features, a positive relationship was observed between normalized peak intensity and peak area (*p* < 0.05), such as I1156 and A1156. The increase in peak area contributed to a significant rise in the peak area ratio in the Raman spectra of different OS samples. The Raman feature differences in binary blended olive-rapeseed oils (OR) and olive-corn oils (OC) are displayed using HCA and CA in S. Fig. 3 and S. Fig. 4, respectively. These differences further demonstrate how varying oil blends influence Raman spectral patterns, which could be critical for detecting adulteration or confirming the authenticity of olive oil blends in commercial products. Overall, the olive oil content in binary OS blends causes significant changes in Raman peak features at 1156, 1265, 1526, and 1658 cm^-1^.

### Discrimination of oil samples using machine learning

Machine learning algorithms, including SVM, PLS-DA, RF, and XGBoost, were investigated to classify ten samples, including four pure oils, three binary blends, and three ternary blends, based on Raman features such as peak intensity (I), peak area (A), peak area ratio (R), full width at half maximum (F), and all features (ALL). The classification results, in terms of accuracy, are visualized through multiple confusion matrices, as shown in Fig. 4. Based on the misclassification results, RF and XGBoost models demonstrate superior performance in identifying multiple oil classes, including both pure and blended oils, when using all Raman features, highlighting the importance of feature selection in model classification. This suggests that RF and XGBoost models are more robust in handling complex data with multiple features, making them particularly effective for classifying heterogeneous oil samples. The importance of feature selection is further emphasized, as using a comprehensive set of Raman features improves the model’s ability to distinguish between oil types with high accuracy. The confusion matrix is derived from the model classification on the independent test set of a single stratified split. The performance should therefore be interpreted with the understanding that it may vary with different data partitions.

**Fig. 5:**
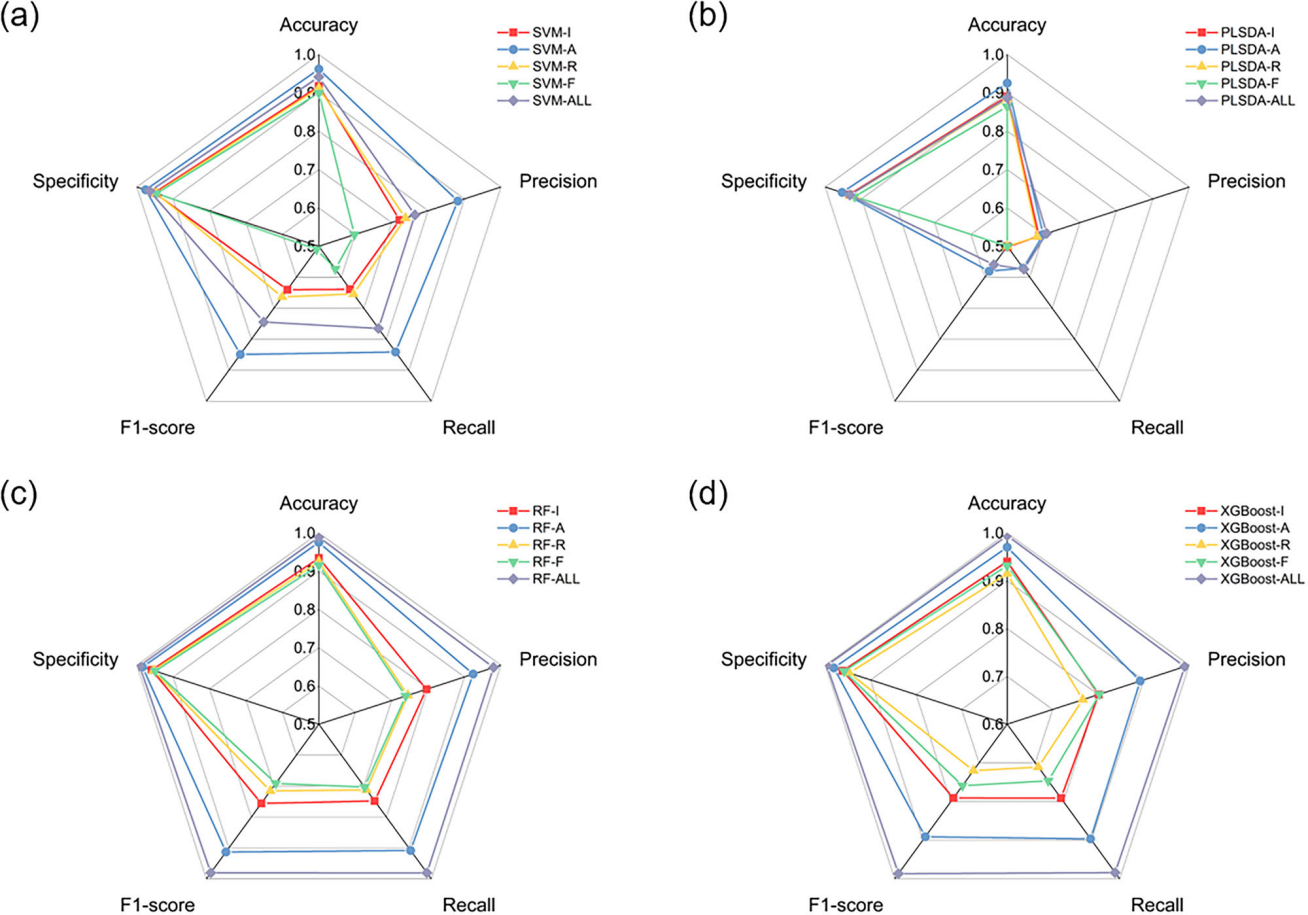
Performance of machine learning models based on different Raman features for the classification of pure and blended oils. Evaluation metrics of **a** SVM, **b** PLS-DA, **c** RF, and **d** XGBoost.

**Fig. 6:**
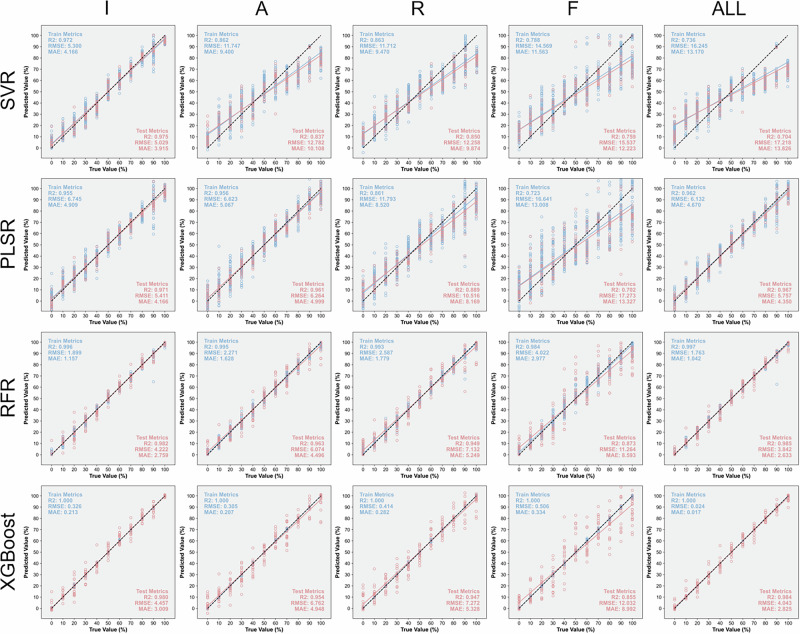
Representative regression plots of machine learning models for binary blended olive-sunflower seed oils (OS) based on various Raman features.

Furthermore, Fig. 5 shows the model evaluation for SVM, PLS-DA, RF, and XGBoost in classifying oil samples using various Raman features as inputs. S. Table 1 provides relevant evaluation metrics, including accuracy, precision, recall, F1-score, and specificity. A 5-fold cross-validation analysis is also shown in S. Table 2. The radar chart indicates that the best-performing models, RF and XGBoost, achieve the highest metrics when all Raman features are used as inputs, followed by peak area. SVM also shows good performance when using the peak area of characteristic bands. Although Raman spectra contain abundant information, peak intensity or height is often used as a preliminary feature in chemometrics for food ingredient analysis^27^. However, peak area and ratio, which provide unique spectral data, should also be considered and could potentially become effective inputs for Raman spectroscopy applications in edible oil authentication^10,28^. The RF model (accuracy = 98.9%, precision = 98.0%, recall = 98.0%, F1-score = 98.0%, specificity = 99.2%) and XGBoost model (accuracy = 99.4%, precision = 99.0%, recall = 98.4%, F1-score = 98.6%, specificity = 99.6%) based on all Raman features outperform individual inputs, such as peak intensity and area, indicating that these two models are optimal for Raman spectra applications. When using a single Raman feature as input, peak area exhibits superior accuracy and precision, with RF achieving 97.5% accuracy and 92.4% precision, and XGBoost achieving 97.0% accuracy and 89.2% precision. A comparative study of different models using the same Raman feature is presented in S. Fig. 6. Both RF and XGBoost demonstrate superiority over SVM and PLS-DA, regardless of the Raman feature input. The performance discrepancies among models primarily stem from differences in their capacity to model nonlinear correlations within high-dimensional spectral data. RF and XGBoost effectively capture complex feature interactions and exhibit excellent generalization ability, whereas SVM and PLS-DA are limited by their linear assumptions, resulting in reduced sensitivity to subtle spectral variations and lower discriminative power. To mitigate potential class imbalance in oil-type discrimination, stratified sampling and macro-averaged metrics were employed to ensure a fair evaluation across all classes and reduce bias toward larger sample groups.

### Determination of blended olive oils using machine learning

**Fig. 7:**
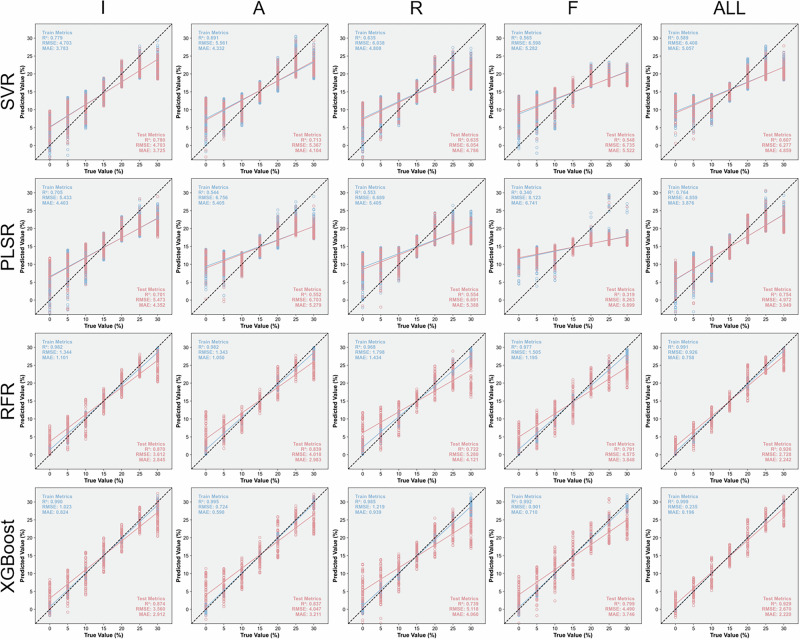
Representative regression plots of machine learning models for ternary blended oils (OSR) composed of olive, sunflower seed, and rapeseed oils based on various Raman features.

**Fig. 8:**
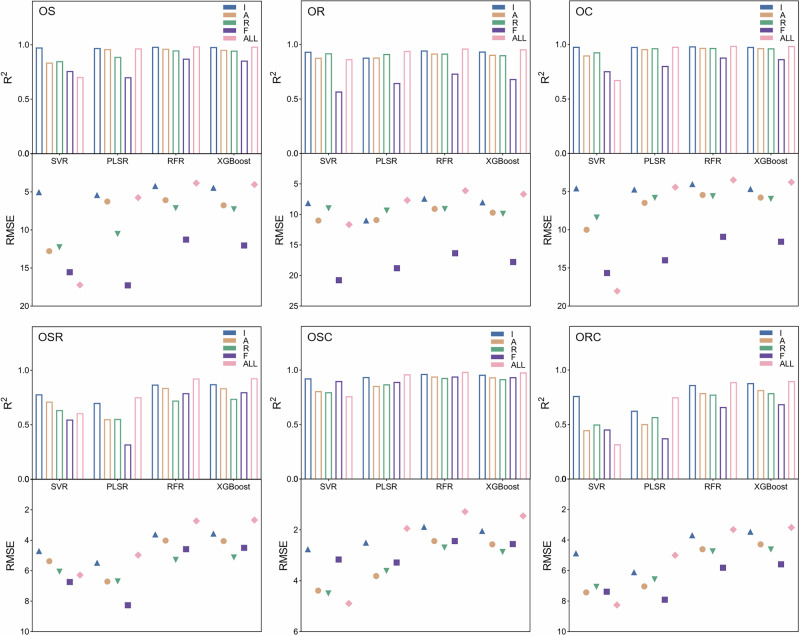
Evaluation of machine learning models for predicting the olive oil content in mixtures. Bars represent the *R*² values, and symbols represent the RMSE values in the combined plot.

For the purpose of determining the blending proportion of olive oil in binary and ternary mixtures, the processed Raman features were further utilized for training and testing chemometric regression models. Four regression algorithms, including SVR, PLSR, RFR, and XGBoost, were employed to estimate the olive oil content based on different Raman features, including I, A, R, F, and ALL. Model performance in quantitative prediction was evaluated using three common statistical indicators, namely root mean square error (RMSE), *R*², and mean absolute error (MAE). Figure 6 presents the prediction results of machine learning models for representative binary blended olive-sunflower seed oils (OS) based on various Raman features. Among the tested models, RFR and XGBoost achieved the highest *R*^2^ values (0.985 and 0.984, respectively) when all Raman features were used, showing predictions that closely fluctuated around the true values and exhibited strong linear correlations. In addition, both models demonstrated relatively lower RMSE and MAE values compared to SVR and PLSR, confirming their superior performance in the quantitative analysis of OS samples. Notably, all four models exhibited improved predictive metrics when using peak intensity as input features compared to other Raman characteristics such as peak area, suggesting that peak intensity provides more discriminative information for concentration estimation. Model regression plots for binary blended olive-rapeseed oils (OR) and olive-corn oils (OC) based on various Raman features are presented in S. Fig. 7 and S. Fig. 8, respectively. The detailed evaluation indicators for the prediction performance of all models on binary blended oils are summarized in S. Table 3, and the corresponding 5-fold cross-validation analysis is shown in S. Table 4. The superior performance of RFR and XGBoost can be attributed to their ensemble learning architectures and strong capability in modeling nonlinear and interactive relationships among Raman features. Both algorithms integrate multiple decision trees to reduce variance and enhance generalization, allowing them to capture subtle compositional variations in blended oils. In contrast, SVR and PLSR, which rely on linear or kernel-based assumptions, are less effective in representing complex spectral-concentration correlations, leading to relatively lower prediction accuracy.

**Fig. 9:**
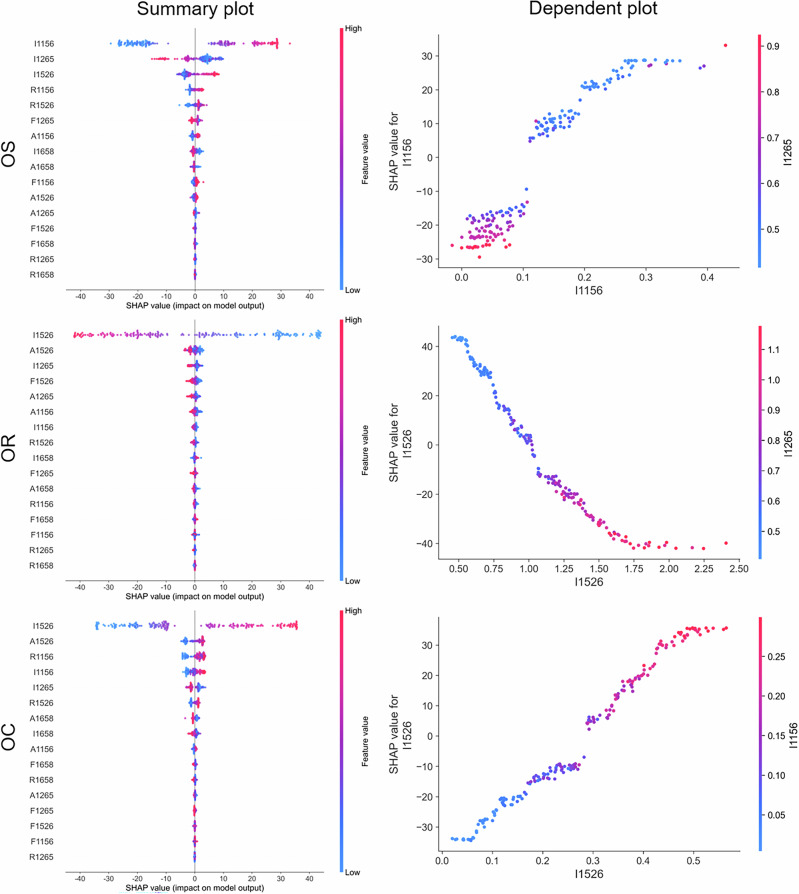
SHAP summary plots of the RF model for binary blended oils, including OS, OR, and OC. SHAP dependence plots show the interaction between the top two Raman features based on peak intensity (I).

**Fig. 10:**
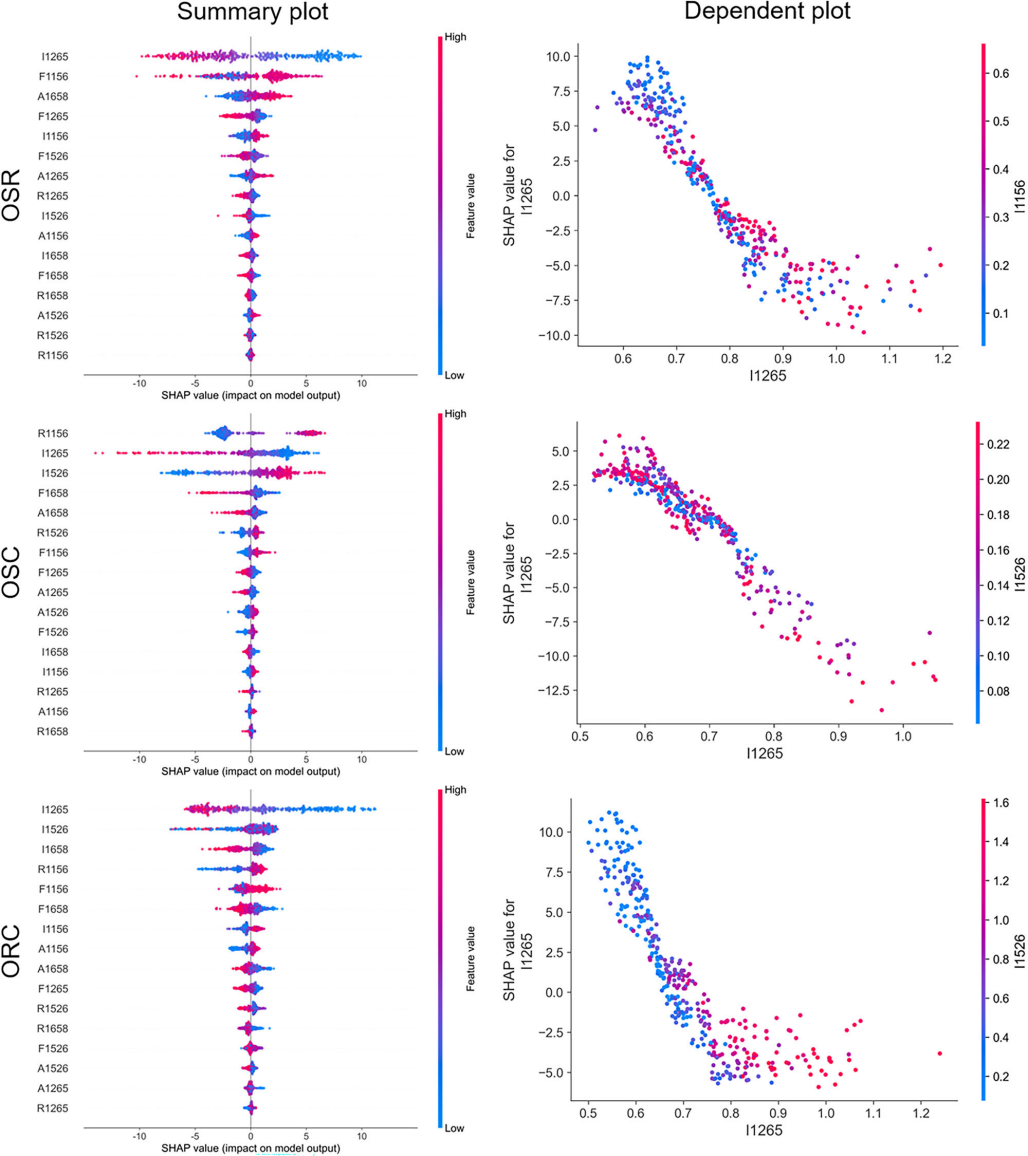
SHAP summary plots of the RF model for ternary blended oils, including OSR, OSC, and ORC. SHAP dependence plots show the interaction between the top two Raman features based on peak intensity (I).

Figure 7 illustrates the prediction results of machine learning models for representative ternary blended oils (OSR) composed of olive, sunflower seed, and rapeseed oils, based on various Raman features. When dealing with complex mixtures synthesized from three different edible oils, the RFR and XGBoost models still exhibit excellent evaluation metrics using all Raman features, achieving *R*^2^ values of 0.926 and 0.929, respectively. Their relatively low RMSE and MAE values further confirm the robustness and reliability of these models in determining the olive oil content in ternary oil samples. These results highlight the capability of ensemble learning algorithms to capture nonlinear spectral interactions among components in complex mixtures, enabling accurate quantification even under overlapping Raman signals. Considering the performance based on single Raman feature inputs, peak intensity emerges as the most effective feature, providing higher prediction accuracy than other spectral descriptors. This finding suggests that Raman intensity conveys richer compositional information than peak area or shift, as it directly reflects the concentration of characteristic molecular vibrations related to unsaturation levels in the blended oils. Model regression plots of ternary blended oils (OSC), composed of olive, sunflower seed, and corn oils, and ternary blended oils (ORC), composed of olive, rapeseed, and corn oils, are presented in S. Fig. 9 and S. Fig. 10, respectively. The detailed evaluation indicators for model performance in predicting ternary blended oils are summarized in S. Table 5, and corresponding 5-fold cross-validation results are provided in S. Table 6. Furthermore, Fig. 8 outlines the evaluation of machine learning models for quantifying olive oil in three binary and three ternary blended oil systems. Among all tested algorithms, the RFR and XGBoost models, which yield high *R*^2^ and low RMSE values, are identified as the most suitable approaches for the quantitative analysis of blended olive oils based on the full set of Raman features. The consistent superiority of these models across both binary and ternary systems indicates their strong generalization ability and potential for practical application in olive oil authentication and quality control.

### Application of SHAP in model explanation for prediction

**Fig. 11:**
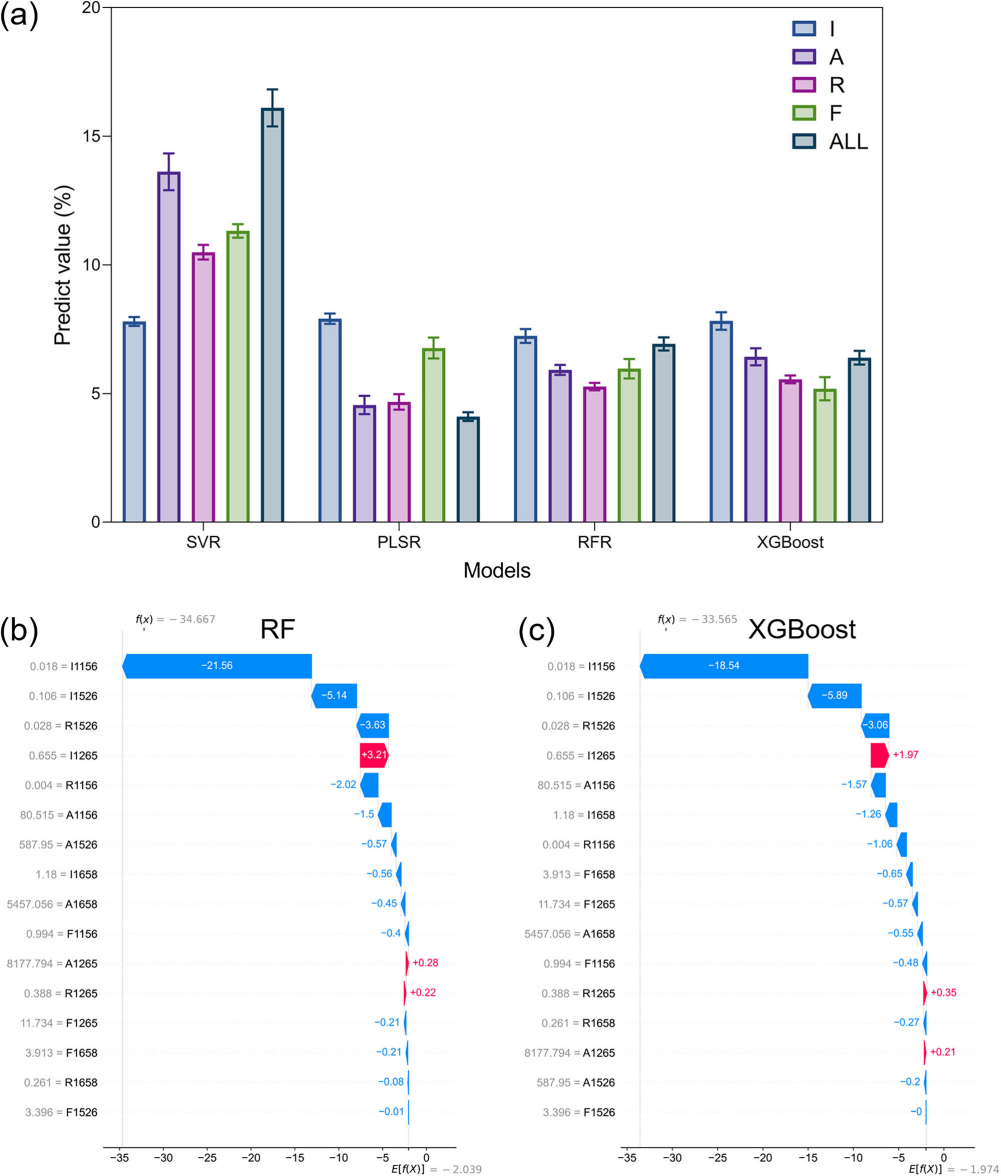
Model validation with commercial BO. **a** Content predictions based on various Raman features, and SHAP waterfall plots of the **b** RF model and **c** XGBoost model.

To interpret the predictions of the two best-performing models, RF and XGBoost, the SHAP algorithm was employed to rank the importance of Raman features and elucidate their interactive effects across various blended oil samples. Figure 9 presents the SHAP analysis for the RF model quantifying olive oil in binary blends. The summary plot for the OS blend indicates that the top two features, I1156 and I1265, exert the most significant influence on the model output. A higher value of I1156 increases the predicted olive oil content, whereas a higher value of I1265 decreases it. This inverse relationship is further illustrated in the dependence plot, showing that low I1156 coupled with high I1265 yields negative SHAP values. These observations are consistent with the distinct compositions of olive and sunflower seed oils^14,20,21^. The strong Raman signals at 1156 and 1526 cm^-1^ originate from β-carotene, which is unique to olive oil, leading to a higher estimated content. Conversely, olive oil is richer in oleic acid, while sunflower seed oil contains more linoleic acid, resulting in weaker Raman intensities at 1265 and 1658 cm^-1^ for olive oil. Thus, high values at I1265 and I1658 indicate a lower proportion of olive oil. Similarly, in the SHAP analysis for OR and OC blends, I1526, attributed to β-carotene was identified as the most contributive feature. Furthermore, the SHAP analysis of the XGBoost model, as depicted in S. Fig. 11, highlighted the importance of I1156 and I1526 in quantifying binary blends, corroborating the findings from the RF model.

The SHAP analysis for ternary blended oils revealed that I1265 is critical to the RF model quantification of olive oil (Fig. 10). Specifically, lower values of I1265 consistently contributed to a higher predicted olive oil content across all ternary blends (OSR, OSC, and ORC). This inverse relationship aligns with the characteristically weaker Raman intensity of olive oil at 1265 cm^-1^ compared to other oils^14,21,29^. This consistency across different ternary systems underscores the generalizability of I1265 as a key spectroscopic marker for olive oil, even in more complex mixtures. The observed phenomenon is potentially associated with its distinct unsaturated fatty acid profile (e.g., oleic acid) and is further supported by data in Fig. 1 and S. Table 10. While the interaction between I1265 and other features (e.g., I1156, I1526) on the SHAP value appeared complex and less distinct, even subtle variations at I1265 proved to be highly important for the quantitative analysis of multicomponent olive oil blends. This suggests that the model successfully captures nuanced compositional changes that may be critical for accurate quantification in complex matrices. This finding was corroborated by the XGBoost model, whose SHAP analysis also underscored the significant impact of I1265 on the predicted olive oil content in ternary systems (S. Fig. 12). The consensus between the RF and XGBoost models not only validates the predictive power of I1265 but also enhances the overall credibility of the SHAP-based interpretation.

### Method validation with commercial BO

To validate the practical applicability of the models, five different commercial blended oil samples comprising other edible oils and olive oil at a 95:5 ratio were quantitatively analyzed. Strikingly, the prediction results from the XGBoost (6.3 ± 1.7%) and RF (6.9 ± 1.6%) models, when using all Raman features, were in close agreement with the actual value (5%), significantly outperforming SVR and PLSR (Fig. 11a). The high accuracy achieved by both RF and XGBoost on commercial samples, which inherently contain complex matrix effects absent in prepared laboratory samples, strongly validates the robustness and practical potential of the proposed analytical framework. Notably, the prediction from the PLSR model (4.1 ± 1.1%) was slightly lower, highlighting that conventional linear models may struggle with the nuanced spectral contributions in such complex mixtures. These findings serve to emphasize the paramount importance of model selection to a certain degree. Furthermore, the significant differences in predictions resulting from different feature sets suggest that the optimal fingerprint of Raman features is essential for unlocking the full potential of the spectroscopy technique for quantitative analysis. While the five commercial blended oil samples provided a meaningful real-world test, their small size and limited concentration coverage constrain statistical power and leave the quantitative accuracy partially unverified across the full gradient. Future work should include a larger and more diverse external set to rigorously assess robustness. Finally, SHAP analysis of the RF and XGBoost models on the commercial samples (Fig. 11b, c) revealed consistent feature importance rankings, with I1156, I1526, and I1265 being the most influential. The stability of this key feature set across both models and its successful application to a real-world sample greatly enhances the credibility of our interpretable machine learning approach and pinpoints the most reliable spectral markers for future applications.

In conclusion, this study establishes a novel Raman spectroscopy framework integrated with machine learning for the authentication and quantification of olive oil in blended products. By analyzing four pure oils and six custom-blended samples (binary and ternary), key spectral features, including peak intensity, peak area, area ratio, and full width at half maximum, were identified as effective discriminators. Multivariate analysis (HCA and CA) not only visualized the sample clustering but also revealed feature trends correlated with olive oil content. Among the chemometric models evaluated, tree-based ensemble methods (RF and XGBoost) demonstrated superior performance over SVM and PLS in both classification and regression tasks. Notably, the Raman peak area was found to be a highly effective alternative to the conventional use of peak intensity, potentially offering greater robustness. The innovative application of the SHAP algorithm provided critical interpretability, identifying I1156, I1526, and I1265 as the most impactful features for prediction, which aligns with the unique biochemical composition of olive oil (e.g., β-carotene and fatty acid profile). The successful quantification of olive oil in various commercial blends further validated the practical applicability and accuracy of our approach.

While the proposed method shows significant promise, the scope of this study, particularly the limited number of ternary blends, suggests a need for validation with a larger and more diverse sample set encompassing different olive varieties, geographical origins, and adulterants to ensure global applicability. Future work will focus on expanding the spectral library and translating the optimized models for use with portable Raman spectrometers. This direction holds strong potential for on-site, rapid screening in supply chains, enhancing quality control and combating fraud in the edible oil industry. The findings underscore the value of combining spectroscopic fingerprints with explainable machine learning for non-destructive food analysis.

## Methods

### Sample preparation

Forty types of edible vegetable oils from various brands and manufacturers were purchased from local markets in Wuhan, China, and the corresponding label information is provided in S. Table 7. Four types of pure oils are included: olive oil (OO), sunflower seed oil (SO), rapeseed oil (RO), and corn oil (CO). All olive oils used are extra virgin olive oil. Five commercial blended oils (BO), composed of other edible oils and olive oil in a 95:5 volume ratio, were also used as the reference for method validation. As shown in S. Table 8, three types of binary blends, including olive-sunflower seed oil (OS), olive-rapeseed oil (OR), and olive-corn oil (OC), were prepared with olive oil content ranging from 0% to 100% (v/v) in 10% increments, resulting in a total of 11 mixed samples for each blend. Five sets of parallel samples were prepared for each binary mixture by blending OO1-OO5 with other edible oils (e.g., SO1-SO5, RO1-RO5, CO1-CO5), respectively. Additionally, in S. Table 9, three types of ternary blends, including olive-sunflower seed-rapeseed oil (OSR), olive-sunflower seed-corn oil (OSC), and olive-rapeseed-corn oil (ORC), were prepared with olive oil content ranging from 0% to 30% (v/v) in 5% increments, producing a total of 35 mixed samples for each adulteration, based on the composition of the other two edible oils. Three sets of parallel samples were prepared for each ternary mixture by blending OO1-OO3 with other edible oils (e.g., SO1-SO3, RO1-RO3, CO1-CO3), respectively.

For sample preparation, the corresponding volume of pure oils was pipetted into dry, clean beakers and thoroughly mixed using an ultrasonic oscillator (DL-480E, Shanghai Five Phase Instrumentation Co., Ltd., China). The blended oils, with a final total volume of 5 mL, were then stored in 10 mL glass vials at 4 °C in a refrigerator for subsequent analysis. A 1 mL aliquot of each oil sample was transferred to a quartz cuvette for Raman spectroscopy measurements. All oil samples were prepared in triplicate and analyzed within three days of preparation.

### Raman measurements and data treatment

Raman spectra from different oil samples were acquired using a microscopic Raman spectrometer (MR-3CHANNELS, Shanghai Oceanhood Technology Co., Ltd., Shanghai, China). A 532 nm laser with 100 mW power, a line width of less than 0.2 nm, and a 10x optical objective lens were applied to obtain Raman spectra in the range of 200 to 3600 cm^-1^, with a spectral resolution of 16 cm^-1^. All measurements were performed at room temperature with an integration time of 1 s, and each oil sample was scanned three times to obtain an averaged spectrum. The Raman laser was positioned at three randomly selected points on the surface of the quartz cuvette, and ten Raman spectra were collected at each position. In total, 90 Raman spectra were obtained for each class of oil samples, resulting in 46,800 spectra across all studied samples.

The peak at 884 cm^-1^, corresponding to ethanol, was used for the calibration of Raman shifts. Preprocessing steps such as baseline correction and denoising^30^ were applied to eliminate fluorescence background interference and instrument noise, thereby improving the signal-to-noise ratio of the raw Raman spectra. In the Raman spectra of pure and blended oils, several features, including normalized peak intensity (I), peak area (A), peak area ratio (R), and full width at half maximum (F), were extracted at specific Raman shifts, such as 1156, 1265, 1526, and 1658 cm^-1^. These values are recorded in S. Table 10. The peak at 1440 cm^-1^, attributed to the deformation vibration of the –CH₂ group, was selected to normalize the Raman spectra and correct for spectral intensity differences^19,20^. The normalized peak intensity was calculated as the ratio of the characteristic peak intensity to the intensity at 1440 cm^-1^. The peak area was estimated as the area enclosed by the characteristic peak and the baseline on the spectrogram, while the peak area ratio was defined as the ratio of the characteristic peak area to the total area of four main peaks^10^. Full width at half maximum was determined by the distance between the two points of the characteristic peak at half the peak height^31,32^. The area under the characteristic spectral peaks was determined via numerical integration. A linear baseline was established for each peak by connecting manually selected start and end points in the adjacent valley regions. After baseline subtraction, the net peak area was calculated by integrating the signal intensity across the peak range using the trapezoidal rule implemented in MATLAB R2021a. For overlapping spectral features, peak deconvolution was performed using the Gaussian-Lorentzian (Voigt) peak fitting function prior to individual area calculation.

### Machine learning

In the pure oil samples, HCA and heatmap visualization^33^ were applied based on clustering patterns to reveal key differentiated features associated with oil types. For binary blended oils, HCA was combined with CA^34^ to explore changes in Raman features with increasing olive oil content and examine the relationship between olive oil proportion and various Raman features. Machine learning algorithms, including SVM, PLS-DA, RF, and XGBoost, were employed to discriminate among multiple oil samples, including four pure oils, three binary blends, and three ternary blends, based on different sets of Raman features. These regression models were also used to predict the olive oil mixing ratio in binary and ternary blends. The principles and procedures for these machine learning models were described in the literature^12–15^. For model development, the spectral data from ten types of oil samples, including four pure oils, three binary blends, and three ternary blends, were randomly split at an 8:2 ratio into training and testing sets for classification. The split was stratified to maintain the class distribution (i.e., the proportion of each oil type) across both the training and test sets, ensuring a representative evaluation. Similarly, the spectral data corresponding to each mixture proportion in the three binary and three ternary blended oils were randomly divided at the same 8:2 ratio and used as the training and testing sets for prediction models. A stratified sampling strategy was also applied to both binary and ternary blended oils, covering the full range of their respective mixing ratios. These preprocessed and structured datasets provided a solid foundation for the subsequent development of classification and prediction models. A 5-fold cross-validation strategy was employed to perform internal validation of the models. Five evaluation metrics, including accuracy, precision, recall, F1-score, and specificity, were used to assess the model performance for oil type classification. For the quantification of blended oils, *R*^2^, RMSE, and MAE were used to evaluate model performance. Additionally, the SHAP algorithm^17^ was applied to identify key Raman features that significantly influence model predictions. All data treatments, including machine learning models, HCA, CA, and SHAP algorithms, were performed using Python 3.9 (available at https://www.python.org/), while scientific plotting was accomplished using GraphPad Prism version 9.0.

## Supplementary information

Supplemental Information

## Data Availability

Data will be made available on request.
