# Supplementary material for: Unveiling key peak features for olive oil authentication utilizing Raman spectroscopy and chemometrics
Source: NPJ Sci Food. 2026 Feb 6;10:88. doi: 10.1038/s41538-026-00738-2 (PMC12988142; doi:10.1038/s41538-026-00738-2)
Supplement: Supplementary file 1 — Supplemental Information [file 41538_2026_738_MOESM1_ESM.docx]

**Supplementary Material**

Unveiling key peak features for olive oil authentication utilizing Raman spectroscopy and chemometrics

Yulong Chen^a^, Renjie Shao^b^, Shan Zeng^b,*^, Bing Li^b^, Huanjun Hu^b^

*^a^ College of Medicine and Health Science, Wuhan Polytechnic University, Wuhan 430023, China*

*^b^ School of Mathematics and Computer Science, Wuhan Polytechnic University, Wuhan 430023, China*

**Correspondence to:**

Prof. Shan Zeng, School of Mathematics and Computer Science, Wuhan Polytechnic University, Wuhan 430023, China

E-mail address: zengshan1981@whpu.edu.cn

**S. Figure**


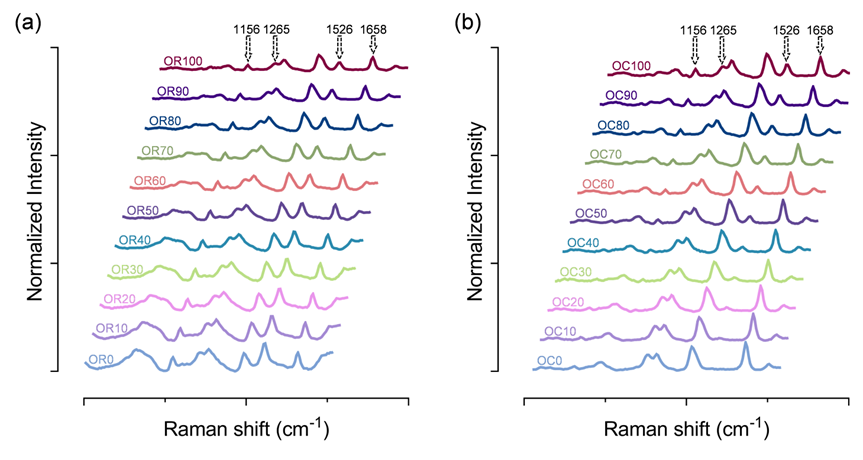


**S. Figure 1**. Raman spectra of binary blended oils including (a) olive-rapeseed oils (OR) and (b) olive-corn oils (OC) at different mixing proportions.


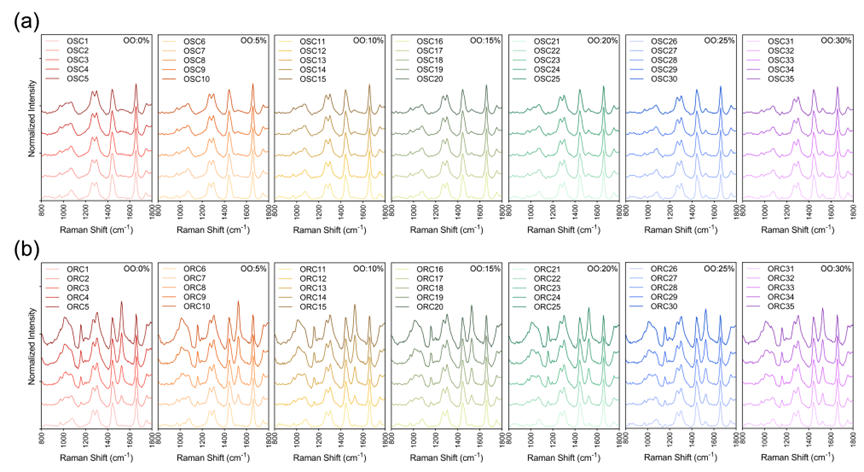


**S. Figure 2**. Raman spectra of ternary blended oils including (a) OSC composed of olive, sunflower seed, and corn oils, and (b) ORC composed of olive, rapeseed, and corn oils at different mixing proportions.

**
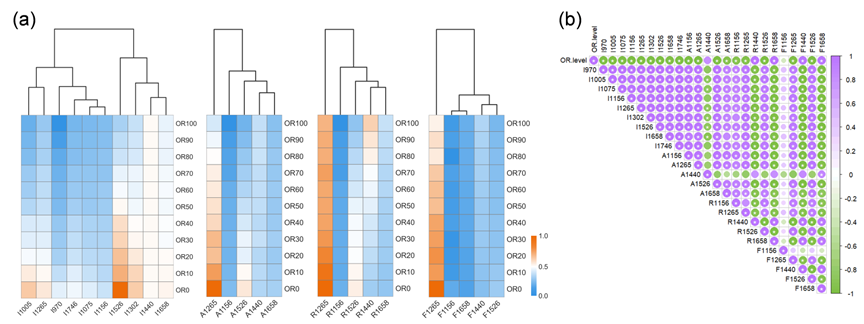
**

**S. Figure 3**. Differences in Raman features of binary blended olive-rapeseed oils (OR). (a) Hierarchical cluster analysis (HCA); (b) Correlation analysis (CA) between olive oil content levels and Raman features.


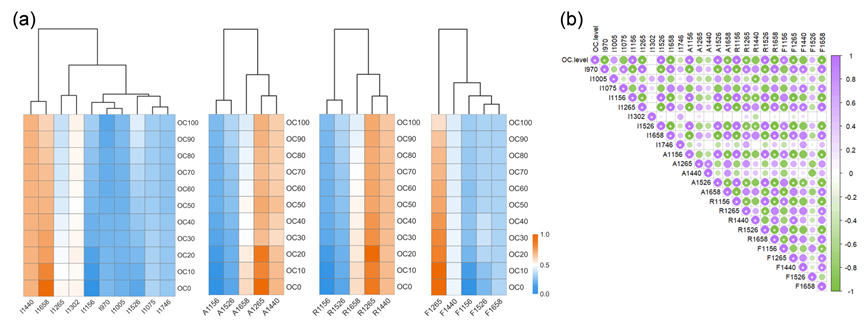


**S. Figure 4**. Differences in Raman features of binary blended olive-corn oils (OC). (a) Hierarchical cluster analysis (HCA); (b) Correlation analysis (CA) between olive oil content levels and Raman features.


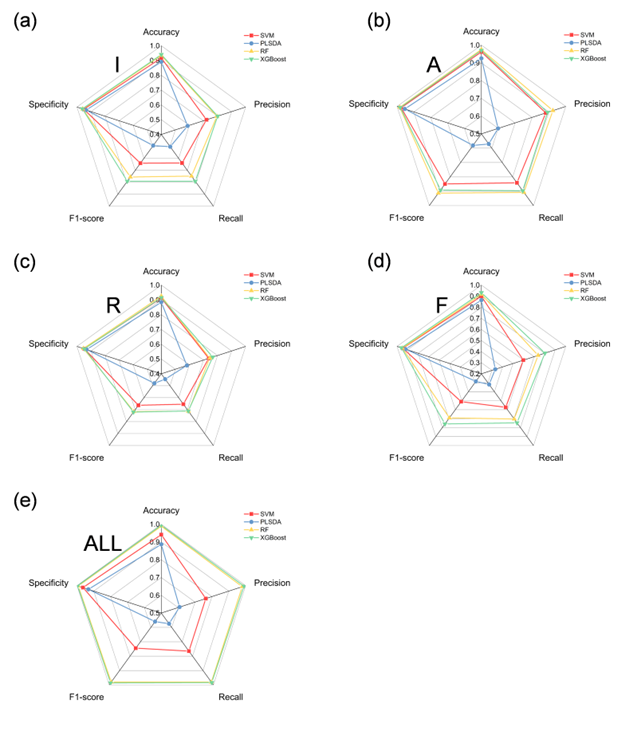


**S. Figure 6**. Performance of machine learning models based on various Raman features for the classification of pure and blended oils: Model input of (a) I, (b) A, (c) R, (d) F, and (e) ALL.


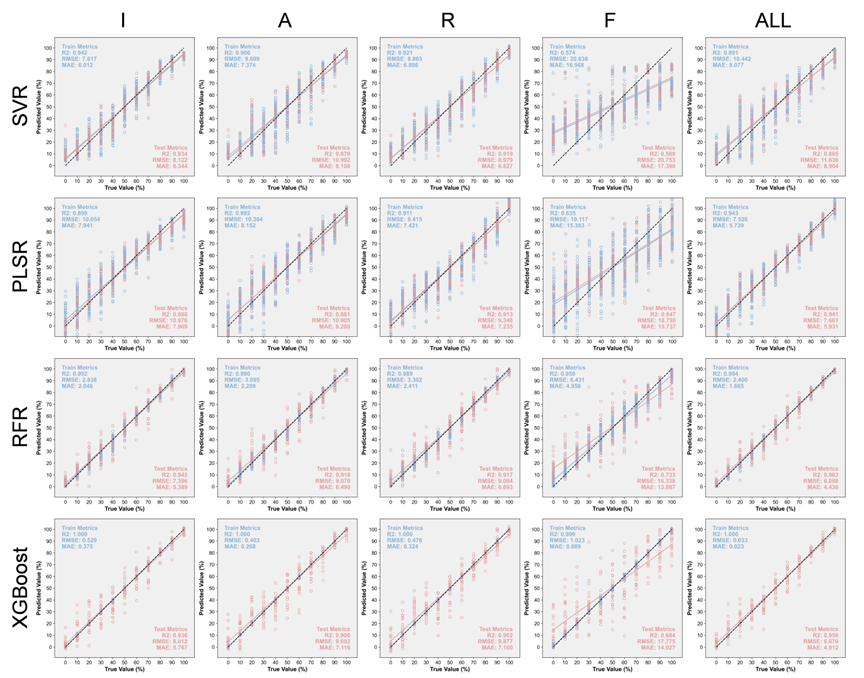


**S. Figure 7**. Representative regression plots of machine learning models for binary blended olive-rapeseed oils (OR) based on various Raman features.


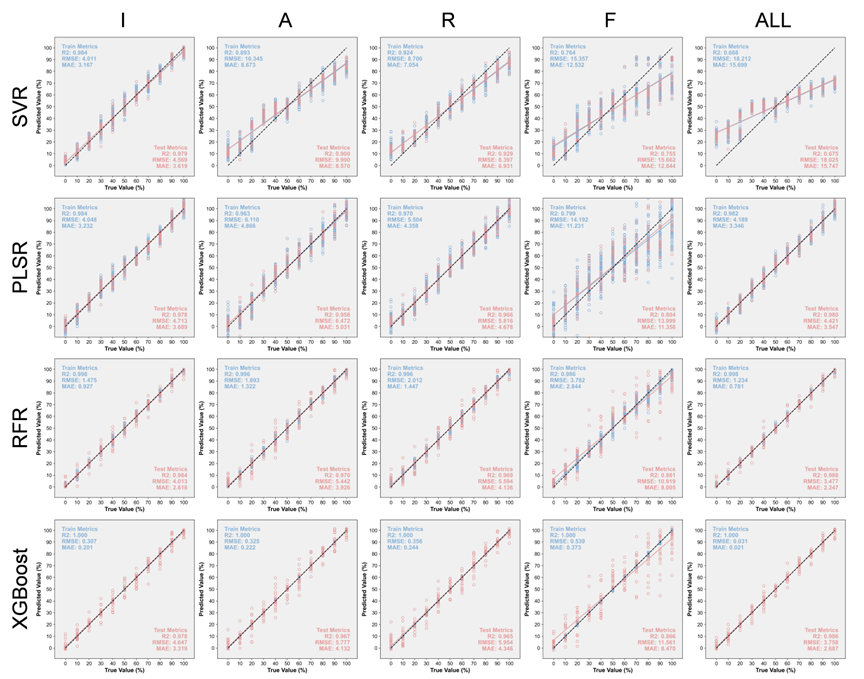


**S. Figure 8**. Representative regression plots of machine learning models for binary blended olive-corn oils (OC) based on various Raman features.


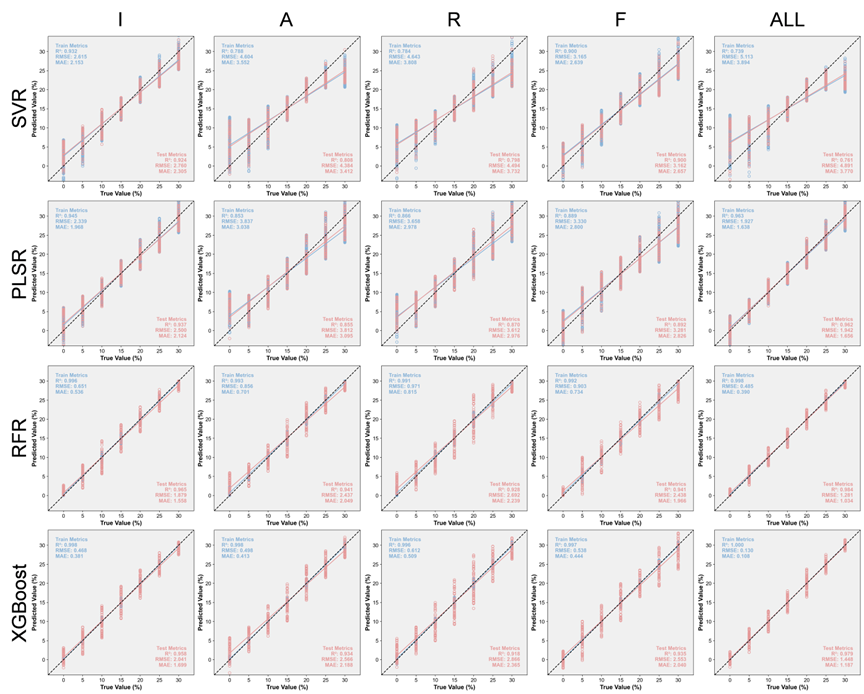


**S. Figure 9**. Representative regression plots of machine learning models for ternary blended oils (OSC) composed of olive, sunflower seed, and corn oils based on various Raman features.


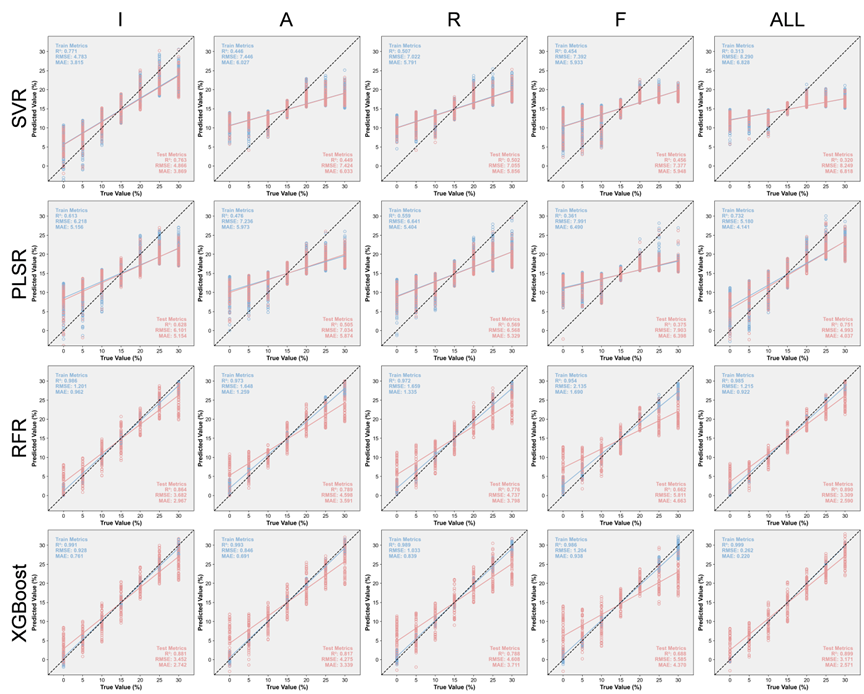


**S. Figure 10**. Representative regression plots of machine learning models for ternary blended oils (ORC) composed of olive, rapeseed, and corn oils based on various Raman features.


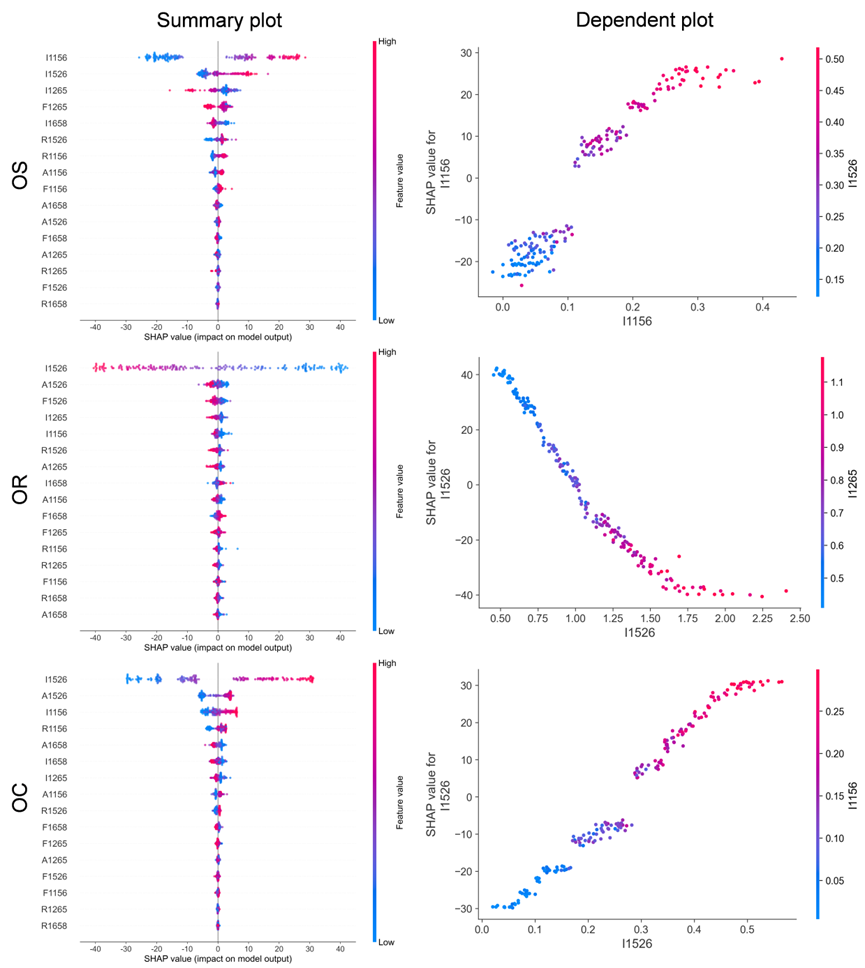


**S. Figure 11**. SHAP summary plots of the XGBoost model for binary blended oils, including OS, OR, and OC. SHAP dependence plots show the interaction between the top two Raman features based on peak intensity (I).


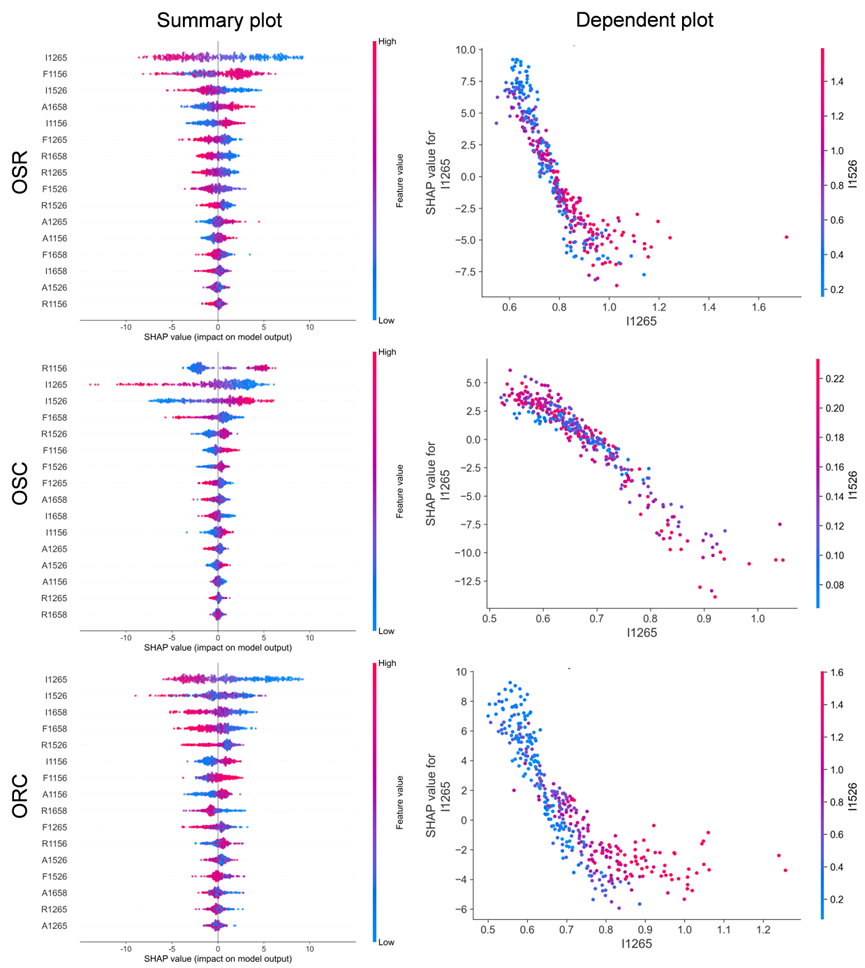


**S. Figure 12**. SHAP summary plots of the XGBoost model for binary blended oils, including OSR, OSC, and ORC. SHAP dependence plots show the interaction between the top two Raman features based on peak intensity (I).

**S. Table**

**S. Table 1.** Evaluation index of machine learning models in the classification.

| Models | Indexes | Inputs | | | | |
| --- | --- | --- | --- | --- | --- | --- |
|  |  | I | A | R | F | ALL |
| SVM | Accuracy | 0.918 | 0.962 | 0.912 | 0.899 | 0.941 |
|  | Precision | 0.722 | 0.882 | 0.738 | 0.598 | 0.764 |
|  | Recall | 0.639 | 0.841 | 0.654 | 0.573 | 0.765 |
|  | F1-score | 0.640 | 0.849 | 0.663 | 0.510 | 0.745 |
|  | Specificity | 0.951 | 0.976 | 0.947 | 0.945 | 0.964 |
| PLSDA | Accuracy | 0.891 | 0.925 | 0.884 | 0.864 | 0.887 |
|  | Precision | 0.587 | 0.599 | 0.582 | 0.332 | 0.608 |
|  | Recall | 0.503 | 0.571 | 0.445 | 0.319 | 0.574 |
|  | F1-score | 0.493 | 0.581 | 0.479 | 0.285 | 0.560 |
|  | Specificity | 0.935 | 0.954 | 0.931 | 0.919 | 0.932 |
| RF | Accuracy | 0.934 | 0.975 | 0.925 | 0.915 | 0.989 |
|  | Precision | 0.796 | 0.924 | 0.744 | 0.738 | **0.980** |
|  | Recall | 0.748 | 0.908 | 0.712 | 0.703 | 0.980 |
|  | F1-score | 0.756 | 0.913 | 0.716 | 0.691 | 0.980 |
|  | Specificity | 0.959 | 0.984 | 0.954 | 0.949 | 0.992 |
| XGBoost | Accuracy | 0.940 | 0.970 | 0.914 | 0.932 | 0.994 |
|  | Precision | 0.801 | 0.892 | 0.766 | 0.803 | **0.990** |
|  | Recall | 0.791 | 0.896 | 0.711 | 0.746 | 0.984 |
|  | F1-score | 0.791 | 0.891 | 0.720 | 0.759 | 0.986 |
|  | Specificity | 0.961 | 0.981 | 0.947 | 0.958 | 0.996 |

**S. Table 2.** Cross-validation analysis of machine learning models in the classification.

| Models | Indexes | Inputs | | | | |
| --- | --- | --- | --- | --- | --- | --- |
|  |  | I | A | R | F | ALL |
| SVM | Accuracy | 0.824 | 0.856 | 0.815 | 0.793 | 0.844 |
|  | Precision | 0.617 | 0.795 | 0.672 | 0.504 | 0.627 |
|  | Recall | 0.582 | 0.728 | 0.619 | 0.493 | 0.634 |
|  | F1-score | 0.601 | 0.734 | 0.594 | 0.456 | 0.628 |
|  | Specificity | 0.835 | 0.846 | 0.822 | 0.818 | 0.859 |
| PLSDA | Accuracy | 0.738 | 0.742 | 0.704 | 0.750 | 0.758 |
|  | Precision | 0.492 | 0.465 | 0.436 | 0.425 | 0.515 |
|  | Recall | 0.416 | 0.448 | 0.368 | 0.413 | 0.502 |
|  | F1-score | 0.403 | 0.451 | 0.395 | 0.396 | 0.489 |
|  | Specificity | 0.734 | 0.753 | 0.725 | 0.755 | 0.746 |
| RF | Accuracy | 0.822 | 0.847 | 0.819 | 0.813 | 0.904 |
|  | Precision | 0.695 | 0.812 | 0.653 | 0.645 | **0.927** |
|  | Recall | 0.635 | 0.796 | 0.645 | 0.629 | 0.918 |
|  | F1-score | 0.624 | 0.789 | 0.637 | 0.624 | 0.905 |
|  | Specificity | 0.839 | 0.853 | 0.825 | 0.820 | 0.913 |
| XGBoost | Accuracy | 0.826 | 0.856 | 0.824 | 0.837 | 0.912 |
|  | Precision | 0.713 | 0.785 | 0.697 | 0.705 | **0.933** |
|  | Recall | 0.705 | 0.794 | 0.675 | 0.699 | 0.919 |
|  | F1-score | 0.698 | 0.779 | 0.663 | 0.651 | 0.926 |
|  | Specificity | 0.834 | 0.858 | 0.816 | 0.823 | 0.925 |

**S. Table 3.** Evaluation index of machine learning models in the prediction of binary blended oils.

| Oils | Indexes | Models | Inputs | | | | |
| --- | --- | --- | --- | --- | --- | --- | --- |
|  |  |  | I | A | R | F | ALL |
| OS | R^2^ | SVR | 0.975 | 0.837 | 0.850 | 0.759 | 0.704 |
|  |  | PLSR | 0.971 | 0.961 | 0.889 | 0.702 | 0.967 |
|  |  | RFR | 0.982 | 0.963 | 0.949 | 0.873 | **0.985** |
|  |  | XGBoost | 0.980 | 0.954 | 0.947 | 0.855 | **0.984** |
|  | RMSE | SVR | 5.029 | 12.782 | 12.258 | 15.537 | 17.218 |
|  |  | PLSR | 5.411 | 6.264 | 10.516 | 17.273 | 5.757 |
|  |  | RFR | 4.222 | 6.074 | 7.132 | 11.264 | 3.842 |
|  |  | XGBoost | 4.457 | 6.762 | 7.272 | 12.032 | 4.043 |
|  | MAE | SVR | 3.915 | 10.108 | 9.874 | 12.223 | 13.826 |
|  |  | PLSR | 4.166 | 4.999 | 8.169 | 13.327 | 4.35 |
|  |  | RFR | 2.759 | 4.496 | 5.249 | 8.593 | 2.633 |
|  |  | XGBoost | 3.009 | 4.948 | 5.328 | 8.992 | 2.825 |
| OR | R^2^ | SVR | 0.934 | 0.879 | 0.919 | 0.569 | 0.865 |
|  |  | PLSR | 0.880 | 0.881 | 0.913 | 0.647 | 0.941 |
|  |  | RFR | 0.945 | 0.918 | 0.917 | 0.733 | **0.963** |
|  |  | XGBoost | 0.936 | 0.906 | 0.902 | 0.684 | **0.956** |
|  | RMSE | SVR | 8.122 | 10.992 | 8.979 | 20.753 | 11.636 |
|  |  | PLSR | 10.976 | 10.905 | 9.348 | 18.798 | 7.661 |
|  |  | RFR | 7.396 | 9.070 | 9.084 | 16.338 | 6.088 |
|  |  | XGBoost | 8.012 | 9.692 | 9.877 | 17.775 | 6.670 |
|  | MAE | SVR | 6.344 | 8.108 | 6.827 | 17.39 | 8.904 |
|  |  | PLSR | 7.969 | 8.28 | 7.235 | 15.737 | 5.931 |
|  |  | RFR | 5.389 | 6.49 | 6.693 | 13.087 | 4.43 |
|  |  | XGBoost | 5.767 | 7.116 | 7.16 | 14.027 | 4.912 |
| OC | R^2^ | SVR | 0.979 | 0.900 | 0.929 | 0.755 | 0.675 |
|  |  | PLSR | 0.978 | 0.958 | 0.966 | 0.804 | 0.980 |
|  |  | RFR | 0.984 | 0.970 | 0.969 | 0.881 | **0.988** |
|  |  | XGBoost | 0.978 | 0.967 | 0.965 | 0.866 | **0.986** |
|  | RMSE | SVR | 4.569 | 9.990 | 8.397 | 15.662 | 18.025 |
|  |  | PLSR | 4.713 | 6.472 | 5.816 | 13.999 | 4.421 |
|  |  | RFR | 4.013 | 5.442 | 5.594 | 10.919 | 3.477 |
|  |  | XGBoost | 4.647 | 5.777 | 5.954 | 11.561 | 3.758 |
|  | MAE | SVR | 3.619 | 8.570 | 6.931 | 12.844 | 15.747 |
|  |  | PLSR | 3.689 | 5.031 | 4.678 | 11.356 | 3.547 |
|  |  | RFR | 2.618 | 3.926 | 4.136 | 8.005 | 2.247 |
|  |  | XGBoost | 3.319 | 4.132 | 4.346 | 8.470 | 2.687 |

**S. Table 4.** Cross-validation analysis of machine learning models in the prediction of binary blended oils.

| Oils | Indexes | Models | Inputs | | | | |
| --- | --- | --- | --- | --- | --- | --- | --- |
|  |  |  | I | A | R | F | ALL |
| OS | R^2^ | SVR | 0.826 | 0.747 | 0.765 | 0.719 | 0.657 |
|  |  | PLSR | 0.813 | 0.816 | 0.782 | 0.638 | 0.829 |
|  |  | RFR | 0.835 | 0.832 | 0.824 | 0.754 | **0.914** |
|  |  | XGBoost | 0.829 | 0.804 | 0.818 | 0.746 | **0.908** |
|  | RMSE | SVR | 13.891 | 15.653 | 14.658 | 14.292 | 18.138 |
|  |  | PLSR | 13.455 | 13.684 | 14.912 | 18.688 | 13.667 |
|  |  | RFR | 13.389 | 13.066 | 13.610 | 14.715 | 8.496 |
|  |  | XGBoost | 13.973 | 13.178 | 13.984 | 14.825 | 9.564 |
|  | MAE | SVR | 9.561 | 13.108 | 12.336 | 14.531 | 15.894 |
|  |  | PLSR | 10.623 | 10.065 | 13.948 | 16.064 | 9.106 |
|  |  | RFR | 8.612 | 8.984 | 9.462 | 14.987 | 6.478 |
|  |  | XGBoost | 9.984 | 9.879 | 10.745 | 14.562 | 7.793 |
| OR | R^2^ | SVR | 0.789 | 0.643 | 0.829 | 0.506 | 0.748 |
|  |  | PLSR | 0.762 | 0.759 | 0.796 | 0.523 | 0.801 |
|  |  | RFR | 0.734 | 0.806 | 0.823 | 0.664 | **0.889** |
|  |  | XGBoost | 0.715 | 0.835 | 0.811 | 0.593 | **0.872** |
|  | RMSE | SVR | 15.064 | 18.293 | 13.891 | 21.354 | 15.289 |
|  |  | PLSR | 15.346 | 15.809 | 14.210 | 20.623 | 13.061 |
|  |  | RFR | 15.859 | 13.478 | 13.563 | 15.784 | 9.865 |
|  |  | XGBoost | 16.351 | 13.386 | 13.138 | 20.912 | 9.917 |
|  | MAE | SVR | 13.284 | 18.264 | 8.974 | 18.164 | 13.684 |
|  |  | PLSR | 13.348 | 13.946 | 13.235 | 17.827 | 9.740 |
|  |  | RFR | 13.633 | 9.358 | 8.985 | 16.785 | 8.342 |
|  |  | XGBoost | 14.197 | 8.289 | 8.865 | 17.578 | 8.465 |
| OC | R^2^ | SVR | 0.824 | 0.862 | 0.819 | 0.672 | 0.598 |
|  |  | PLSR | 0.806 | 0.815 | 0.844 | 0.705 | 0.840 |
|  |  | RFR | 0.836 | 0.838 | 0.828 | 0.782 | **0.892** |
|  |  | XGBoost | 0.847 | 0.823 | 0.820 | 0.743 | **0.876** |
|  | RMSE | SVR | 12.863 | 9.165 | 13.492 | 15.613 | 19.893 |
|  |  | PLSR | 13.698 | 13.346 | 10.735 | 13.564 | 10.531 |
|  |  | RFR | 11.561 | 11.603 | 12.796 | 12.174 | 9.136 |
|  |  | XGBoost | 10.238 | 12.777 | 12.948 | 13.361 | 9.289 |
|  | MAE | SVR | 9.860 | 8.384 | 10.847 | 14.894 | 15.751 |
|  |  | PLSR | 10.956 | 10.837 | 8.765 | 13.765 | 9.804 |
|  |  | RFR | 9.641 | 9.856 | 9.533 | 11.056 | 8.354 |
|  |  | XGBoost | 9.623 | 9.642 | 9.898 | 12.780 | 8.467 |

**S. Table 5.** Evaluation index of machine learning models in the prediction of ternary blended oils.

| Oils | Indexes | Models | Inputs | | | | |
| --- | --- | --- | --- | --- | --- | --- | --- |
|  |  |  | I | A | R | F | ALL |
| OSR | R^2^ | SVR | 0.780 | 0.713 | 0.635 | 0.548 | 0.607 |
|  |  | PLSR | 0.701 | 0.552 | 0.554 | 0.319 | 0.754 |
|  |  | RFR | 0.870 | 0.839 | 0.722 | 0.791 | **0.926** |
|  |  | XGBoost | 0.874 | 0.837 | 0.739 | 0.799 | **0.929** |
|  | RMSE | SVR | 4.703 | 5.367 | 6.054 | 6.735 | 6.277 |
|  |  | PLSR | 5.473 | 6.703 | 6.691 | 8.263 | 4.972 |
|  |  | RFR | 3.612 | 4.018 | 5.280 | 4.575 | 2.728 |
|  |  | XGBoost | 3.560 | 4.047 | 5.118 | 4.490 | 2.670 |
|  | MAE | SVR | 3.725 | 4.104 | 4.786 | 5.522 | 4.859 |
|  |  | PLSR | 4.352 | 5.279 | 5.388 | 6.899 | 3.949 |
|  |  | RFR | 2.845 | 2.983 | 4.121 | 3.848 | 2.242 |
|  |  | XGBoost | 2.912 | 3.211 | 4.060 | 3.746 | 2.220 |
| OSC | R^2^ | SVR | 0.924 | 0.808 | 0.798 | 0.900 | 0.761 |
|  |  | PLSR | 0.937 | 0.855 | 0.870 | 0.892 | 0.962 |
|  |  | RFR | 0.965 | 0.941 | 0.928 | 0.941 | **0.984** |
|  |  | XGBoost | 0.958 | 0.934 | 0.918 | 0.935 | **0.979** |
|  | RMSE | SVR | 2.760 | 4.384 | 4.494 | 3.162 | 4.891 |
|  |  | PLSR | 2.500 | 3.812 | 3.612 | 3.281 | 1.942 |
|  |  | RFR | 1.879 | 2.437 | 2.692 | 2.438 | 1.281 |
|  |  | XGBoost | 2.041 | 2.566 | 2.866 | 2.553 | 1.448 |
|  | MAE | SVR | 2.305 | 3.412 | 3.732 | 2.657 | 3.770 |
|  |  | PLSR | 2.124 | 3.095 | 2.976 | 2.826 | 1.656 |
|  |  | RFR | 1.558 | 2.049 | 2.239 | 1.966 | 1.034 |
|  |  | XGBoost | 1.699 | 2.188 | 2.365 | 2.040 | 1.187 |
| ORC | R^2^ | SVR | 0.763 | 0.449 | 0.502 | 0.456 | 0.320 |
|  |  | PLSR | 0.628 | 0.505 | 0.569 | 0.375 | 0.751 |
|  |  | RFR | 0.864 | 0.789 | 0.776 | 0.662 | **0.890** |
|  |  | XGBoost | 0.881 | 0.817 | 0.788 | 0.688 | **0.899** |
|  | RMSE | SVR | 4.866 | 7.424 | 7.055 | 7.377 | 8.249 |
|  |  | PLSR | 6.101 | 7.034 | 6.568 | 7.903 | 4.993 |
|  |  | RFR | 3.682 | 4.598 | 4.737 | 5.811 | 3.309 |
|  |  | XGBoost | 3.452 | 4.275 | 4.608 | 5.585 | 3.171 |
|  | MAE | SVR | 3.869 | 6.033 | 5.856 | 5.948 | 6.818 |
|  |  | PLSR | 5.154 | 5.874 | 5.329 | 6.398 | 4.037 |
|  |  | RFR | 2.967 | 3.591 | 3.798 | 4.663 | 2.590 |
|  |  | XGBoost | 2.742 | 3.339 | 3.711 | 4.370 | 2.571 |

**S. Table 6.** Cross-validation analysis of machine learning models in the prediction of ternary blended oils.

| Oils | Indexes | Models | Inputs | | | | |
| --- | --- | --- | --- | --- | --- | --- | --- |
|  |  |  | I | A | R | F | ALL |
| OSR | R^2^ | SVR | 0.621 | 0.607 | 0.510 | 0.434 | 0.562 |
|  |  | PLSR | 0.583 | 0.453 | 0.427 | 0.286 | 0.634 |
|  |  | RFR | 0.716 | 0.724 | 0.638 | 0.558 | **0.857** |
|  |  | XGBoost | 0.729 | 0.695 | 0.619 | 0.599 | **0.861** |
|  | RMSE | SVR | 6.186 | 6.594 | 7.831 | 8.617 | 6.498 |
|  |  | PLSR | 7.645 | 8.432 | 8.507 | 9.227 | 6.321 |
|  |  | RFR | 5.948 | 5.661 | 6.109 | 7.523 | 3.653 |
|  |  | XGBoost | 5.570 | 5.978 | 6.275 | 6.386 | 3.426 |
|  | MAE | SVR | 4.651 | 4.910 | 5.946 | 6.534 | 5.870 |
|  |  | PLSR | 5.722 | 6.652 | 6.157 | 8.368 | 4.583 |
|  |  | RFR | 4.768 | 4.636 | 4.491 | 5.189 | 2.846 |
|  |  | XGBoost | 4.545 | 4.947 | 4.543 | 5.498 | 2.775 |
| OSC | R^2^ | SVR | 0.819 | 0.697 | 0.683 | 0.754 | 0.658 |
|  |  | PLSR | 0.804 | 0.684 | 0.734 | 0.769 | 0.847 |
|  |  | RFR | 0.833 | 0.846 | 0.839 | 0.856 | **0.884** |
|  |  | XGBoost | 0.827 | 0.795 | 0.825 | 0.835 | **0.896** |
|  | RMSE | SVR | 3.679 | 6.503 | 6.772 | 5.725 | 6.891 |
|  |  | PLSR | 3.728 | 6.764 | 5.832 | 5.435 | 3.752 |
|  |  | RFR | 3.425 | 3.663 | 3.418 | 3.223 | 3.158 |
|  |  | XGBoost | 3.512 | 3.289 | 3.506 | 3.486 | 3.173 |
|  | MAE | SVR | 3.486 | 4.578 | 4.854 | 4.758 | 4.724 |
|  |  | PLSR | 3.553 | 4.134 | 4.376 | 4.562 | 3.535 |
|  |  | RFR | 3.179 | 3.395 | 3.239 | 3.409 | 2.467 |
|  |  | XGBoost | 3.304 | 4.232 | 3.365 | 3.270 | 2.382 |
| ORC | R^2^ | SVR | 0.651 | 0.406 | 0.468 | 0.433 | 0.285 |
|  |  | PLSR | 0.498 | 0.465 | 0.515 | 0.358 | 0.647 |
|  |  | RFR | 0.724 | 0.647 | 0.716 | 0.562 | **0.843** |
|  |  | XGBoost | 0.763 | 0.739 | 0.745 | 0.678 | **0.850** |
|  | RMSE | SVR | 6.275 | 9.381 | 8.790 | 8.782 | 9.989 |
|  |  | PLSR | 8.658 | 8.732 | 7.764 | 9.734 | 6.245 |
|  |  | RFR | 5.669 | 6.378 | 5.755 | 7.453 | 3.674 |
|  |  | XGBoost | 5.356 | 5.467 | 5.498 | 6.539 | 3.563 |
|  | MAE | SVR | 4.856 | 5.357 | 5.673 | 5.678 | 8.437 |
|  |  | PLSR | 5.047 | 5.196 | 4.945 | 7.223 | 4.965 |
|  |  | RFR | 3.655 | 4.328 | 3.863 | 4.534 | 3.258 |
|  |  | XGBoost | 3.598 | 3.310 | 3.239 | 4.046 | 3.189 |

**S. Table 7.** Information of edible vegetable oils in the study.

| Label | Types | Brands | Producers | Origins | Best by |
| --- | --- | --- | --- | --- | --- |
| OO1 | Olive oil | Extra virgin olive oil Val Di Mazara | Walmart | Italy | 2026-04-24 |
| OO2 | Olive oil | Extra virgin olive oil OLITALIA | Walmart | Italy | 2027-01-07 |
| OO3 | Olive oil | Extra virgin olive oil Olivoila | Yihaikerry Food Group Co., Ltd. | Spain | 2026-08-09 |
| OO4 | Olive oil | Spanish OLIVE OIL BETIS | Torres Y Ribelles, S.A. | Spain | 2026-01-18 |
| OO5 | Olive oil | Extra virgin olive oil VerdOliva | Shandong Luhua Group Co., Ltd. | Spain | 2025-10-24 |
| OO6 | Olive oil | Jingmi Extra virgin olive oil | Golden Sun Grain and Oil Co., Ltd. | Spain | 2026-09-21 |
| OO7 | Olive oil | MUELOLIVA Extra virgin olive oil | MUELOLIVA Minerva S.L. | Spain | 2026-08-25 |
| OO8 | Olive oil | Oleo Bella Extra virgin olive oil | Aceites Maeva, S.L.U | Spain | 2027-11-29 |
| OO9 | Olive oil | Olis Bargalló Extra virgin olive oil | OLIS BARGALLO SA | Spain | 2027-04-01 |
| OO10 | Olive oil | MARFUGA OLIO EXTRAVERGINE DI OLIVA ITALIANO | Piaodaorenzhi Beijing Commerce and Trade Co., Ltd. | Italy | 2027-04-10 |
| SO1 | Sunflower seed oil | Arawana brand Sunflower seed oil | Yihaikerry Food Group Co., Ltd. | China | 2026-09-20 |
| SO2 | Sunflower seed oil | Luhua Squeezing Sunflower seed oil | Shandong Luhua Group Co., Ltd. | China | 2025-09-18 |
| SO3 | Sunflower seed oil | Shakia Sunflower seed oil | Wuhan Changjiang Sandgull Vegetable Oil Co., Ltd. | China | 2025-10-24 |
| SO4 | Sunflower seed oil | MIGHTY Sunflower seed oil | STANDARD Food Co., Ltd. | China | 2026-01-02 |
| SO5 | Sunflower seed oil | LONGEVITY FLOWER pressing sunflower seed oil | Longevity Flower Food Group Co., Ltd. | China | 2026-10-16 |
| SO6 | Sunflower seed oil | Fulinmen sunflower seed oil | COFCO Group Co., Ltd. | China | 2027-01-27 |
| SO7 | Sunflower seed oil | Kuiwang sunflower seed oil | Golden Sun Grain and Oil Co., Ltd. | China | 2025-12-20 |
| SO8 | Sunflower seed oil | BEIDAHUANG sunflower seed oil | BEIDAHUANG Agriculture Group Co., Ltd. | China | 2026-11-29 |
| SO9 | Sunflower seed oil | JIUSAN sunflower seed oil | JIUSAN Grain & Oil Industrial Group Co., Ltd. | China | 2026-06-30 |
| SO10 | Sunflower seed oil | Chucui sunflower seed oil | COFCO Group Co., Ltd. | China | 2027-02-10 |
| RO1 | Rapeseed oil | Arawana brand Rapeseed oil | Yihaikerry Food Group Co., Ltd. | China | 2026-07-25 |
| RO2 | Rapeseed oil | Luhua Extra Fragrance Rapeseed oil | Shandong Luhua Group Co., Ltd. | China | 2026-02-09 |
| RO3 | Rapeseed oil | Chang ’an Flower Rapeseed oil | Chang ’an Flower Grain and Oil Co., Ltd. | China | 2026-01-05 |
| RO4 | Rapeseed oil | MIAOPEI Rapeseed oil | Changsha Guitaitai Camellia Technology Development Co., Ltd. | China | 2026-12-15 |
| RO5 | Rapeseed oil | Fulinmen Rapeseed oil | COFCO Group Co., Ltd. | China | 2026-11-28 |
| RO6 | Rapeseed oil | MIGHTY Rapeseed oil | STANDARD Food Co., Ltd. | China | 2026-12-24 |
| RO7 | Rapeseed oil | LONGEVITY FLOWER Rapeseed oil | Longevity Flower Food Group Co., Ltd. | China | 2027-01-13 |
| RO8 | Rapeseed oil | Daodaoquan Rapeseed oil | Hunan Baling Oils & Fats Co., Ltd. | China | 2027-01-29 |
| RO9 | Rapeseed oil | One's Member Rapeseed oil | Jiangsu Jinzhou Grain & Oil Foodstuff Co., Ltd. | China | 2027-08-18 |
| RO10 | Rapeseed oil | Tianfu Rapeseed oil | Sichuan Linghai Oils & Fats High-Quality Grain and Oil Co., Ltd. | China | 2026-10-11 |
| CO1 | Corn oil | Arawana brand Corn oil | Yihaikerry Food Group Co., Ltd. | China | 2026-11-09 |
| CO2 | Corn oil | Luhua Extra Fragrance Corn oil | Shandong Luhua Group Co., Ltd. | China | 2026-02-19 |
| CO3 | Corn oil | MIGHTY Corn oil | STANDARD Food Co., Ltd. | China | 2025-09-08 |
| CO4 | Corn oil | LONGEVITY FLOWER Corn oil | Longevity Flower Food Group Co., Ltd. | China | 2025-11-01 |
| CO5 | Corn oil | Fulinmen Corn oil | COFCO Group Co., Ltd. | China | 2026-03-22 |
| CO6 | Corn oil | JIUSAN Corn oil | JIUSAN Grain & Oil Industrial Group Co., Ltd. | China | 2027-09-13 |
| CO7 | Corn oil | Chucui Corn oil | COFCO Group Co., Ltd. | China | 2026-11-13 |
| CO8 | Corn oil | Knife Corn oil | Shenzhen Nanshun Oils & Fats Co., Ltd. | China | 2026-10-25 |
| CO9 | Corn oil | XIWANG Corn oil | Shandong Xiwang Food Co., Ltd. | China | 2027-04-27 |
| CO10 | Corn oil | MASTER GOLD Corn oil | LDC COMPANY | China | 2026-01-21 |
| BO1 | Blended oil (SO:OO,95:5) | BETIS LAOLEAF Blended oil | Torres Y Ribelles, S.A. | Spain | 2026-03-24 |
| BO2 | Blended oil (SO:OO,95:5) | Sanmark Blended oil | Liaoning Shengmai Industrial Co., Ltd. | China | 2026-09-05 |
| BO3 | Blended oil (SO:OO,95:5) | Kuiwang Blended oil | Golden Sun Grain and Oil Co., Ltd. | China | 2026-03-09 |
| BO4 | Blended oil (CO:OO,95:5) | Arawana brand Blended oil | Yihaikerry Food Group Co., Ltd. | China | 2027-02-19 |
| BO5 | Blended oil (CO:OO,95:5) | Chucui Blended oil | COFCO Group Co., Ltd. | China | 2027-06-30 |

**S. Table 8.** Sample preparation of binary blended oils.

| Label | Sample | Proportion | Label | Sample | Proportion | Label | Sample | Proportion |
| --- | --- | --- | --- | --- | --- | --- | --- | --- |
| OS0 | OO:SO | 0:100 | OR0 | OO:RO | 0:100 | OC0 | OO:CO | 0:100 |
| OS10 | OO:SO | 10:90 | OR10 | OO:RO | 10:90 | OC10 | OO:CO | 10:90 |
| OS20 | OO:SO | 20:80 | OR20 | OO:RO | 20:80 | OC20 | OO:CO | 20:80 |
| OS30 | OO:SO | 30:70 | OR30 | OO:RO | 30:70 | OC30 | OO:CO | 30:70 |
| OS40 | OO:SO | 40:60 | OR40 | OO:RO | 40:60 | OC40 | OO:CO | 40:60 |
| OS50 | OO:SO | 50:50 | OR50 | OO:RO | 50:50 | OC50 | OO:CO | 50:50 |
| OS60 | OO:SO | 60:40 | OR60 | OO:RO | 60:40 | OC60 | OO:CO | 60:40 |
| OS70 | OO:SO | 70:30 | OR70 | OO:RO | 70:30 | OC70 | OO:CO | 70:30 |
| OS80 | OO:SO | 80:20 | OR80 | OO:RO | 80:20 | OC80 | OO:CO | 80:20 |
| OS90 | OO:SO | 90:10 | OR90 | OO:RO | 90:10 | OC90 | OO:CO | 90:10 |
| OS100 | OO:SO | 100:0 | OR100 | OO:RO | 100:0 | OC100 | OO:CO | 100:0 |

**S. Table 9.** Sample preparation of ternary blended oils.

| Label | Sample | Proportion | Label | Sample | Proportion | Label | Sample | Proportion |
| --- | --- | --- | --- | --- | --- | --- | --- | --- |
| OSR1 | OO:SO:RO | 0:0:100 | OSC1 | OO:SO:CO | 0:0:100 | ORC1 | OO:RO:CO | 0:0:100 |
| OSR2 | OO:SO:RO | 0:25:75 | OSC2 | OO:SO:CO | 0:25:75 | ORC2 | OO:RO:CO | 0:25:75 |
| OSR3 | OO:SO:RO | 0:50:50 | OSC3 | OO:SO:CO | 0:50:50 | ORC3 | OO:RO:CO | 0:50:50 |
| OSR4 | OO:SO:RO | 0:75:25 | OSC4 | OO:SO:CO | 0:75:25 | ORC4 | OO:RO:CO | 0:75:25 |
| OSR5 | OO:SO:RO | 0:100:0 | OSC5 | OO:SO:CO | 0:100:0 | ORC5 | OO:RO:CO | 0:100:0 |
| OSR6 | OO:SO:RO | 5:0:95 | OSC6 | OO:SO:CO | 5:0:95 | ORC6 | OO:RO:CO | 5:0:95 |
| OSR7 | OO:SO:RO | 5:25:70 | OSC7 | OO:SO:CO | 5:25:70 | ORC7 | OO:RO:CO | 5:25:70 |
| OSR8 | OO:SO:RO | 5:45:50 | OSC8 | OO:SO:CO | 5:45:50 | ORC8 | OO:RO:CO | 5:45:50 |
| OSR9 | OO:SO:RO | 5:70:25 | OSC9 | OO:SO:CO | 5:70:25 | ORC9 | OO:RO:CO | 5:70:25 |
| OSR10 | OO:SO:RO | 5:95:0 | OSC10 | OO:SO:CO | 5:95:0 | ORC10 | OO:RO:CO | 5:95:0 |
| OSR11 | OO:SO:RO | 10:0:90 | OSC11 | OO:SO:CO | 10:0:90 | ORC11 | OO:RO:CO | 10:0:90 |
| OSR12 | OO:SO:RO | 10:20:70 | OSC12 | OO:SO:CO | 10:20:70 | ORC12 | OO:RO:CO | 10:20:70 |
| OSR13 | OO:SO:RO | 10:45:45 | OSC13 | OO:SO:CO | 10:45:45 | ORC13 | OO:RO:CO | 10:45:45 |
| OSR14 | OO:SO:RO | 10:70:20 | OSC14 | OO:SO:CO | 10:70:20 | ORC14 | OO:RO:CO | 10:70:20 |
| OSR15 | OO:SO:RO | 10:90:0 | OSC15 | OO:SO:CO | 10:90:0 | ORC15 | OO:RO:CO | 10:90:0 |
| OSR16 | OO:SO:RO | 15:0:85 | OSC16 | OO:SO:CO | 15:0:85 | ORC16 | OO:RO:CO | 15:0:85 |
| OSR17 | OO:SO:RO | 15:20:65 | OSC17 | OO:SO:CO | 15:20:65 | ORC17 | OO:RO:CO | 15:20:65 |
| OSR18 | OO:SO:RO | 15:40:45 | OSC18 | OO:SO:CO | 15:40:45 | ORC18 | OO:RO:CO | 15:40:45 |
| OSR19 | OO:SO:RO | 15:60:25 | OSC19 | OO:SO:CO | 15:60:25 | ORC19 | OO:RO:CO | 15:60:25 |
| OSR20 | OO:SO:RO | 15:85:0 | OSC20 | OO:SO:CO | 15:85:0 | ORC20 | OO:RO:CO | 15:85:0 |
| OSR21 | OO:SO:RO | 20:0:80 | OSC21 | OO:SO:CO | 20:0:80 | ORC21 | OO:RO:CO | 20:0:80 |
| OSR22 | OO:SO:RO | 20:20:60 | OSC22 | OO:SO:CO | 20:20:60 | ORC22 | OO:RO:CO | 20:20:60 |
| OSR23 | OO:SO:RO | 20:40:40 | OSC23 | OO:SO:CO | 20:40:40 | ORC23 | OO:RO:CO | 20:40:40 |
| OSR24 | OO:SO:RO | 20:60:20 | OSC24 | OO:SO:CO | 20:60:20 | ORC24 | OO:RO:CO | 20:60:20 |
| OSR25 | OO:SO:RO | 20:80:0 | OSC25 | OO:SO:CO | 20:80:0 | ORC25 | OO:RO:CO | 20:80:0 |
| OSR26 | OO:SO:RO | 25:0:75 | OSC26 | OO:SO:CO | 25:0:75 | ORC26 | OO:RO:CO | 25:0:75 |
| OSR27 | OO:SO:RO | 25:20:55 | OSC27 | OO:SO:CO | 25:20:55 | ORC27 | OO:RO:CO | 25:20:55 |
| OSR28 | OO:SO:RO | 25:35:40 | OSC28 | OO:SO:CO | 25:35:40 | ORC28 | OO:RO:CO | 25:35:40 |
| OSR29 | OO:SO:RO | 25:55:20 | OSC29 | OO:SO:CO | 25:55:20 | ORC29 | OO:RO:CO | 25:55:20 |
| OSR30 | OO:SO:RO | 25:75:0 | OSC30 | OO:SO:CO | 25:75:0 | ORC30 | OO:RO:CO | 25:75:0 |
| OSR31 | OO:SO:RO | 30:0:70 | OSC31 | OO:SO:CO | 30:0:70 | ORC31 | OO:RO:CO | 30:0:70 |
| OSR32 | OO:SO:RO | 30:20:50 | OSC32 | OO:SO:CO | 30:20:50 | ORC32 | OO:RO:CO | 30:20:50 |
| OSR33 | OO:SO:RO | 30:35:35 | OSC33 | OO:SO:CO | 30:35:35 | ORC33 | OO:RO:CO | 30:35:35 |
| OSR34 | OO:SO:RO | 30:50:20 | OSC34 | OO:SO:CO | 30:50:20 | ORC34 | OO:RO:CO | 30:50:20 |
| OSR35 | OO:SO:RO | 30:70:0 | OSC35 | OO:SO:CO | 30:70:0 | ORC35 | OO:RO:CO | 30:70:0 |

**S. Table 10.** Raman features of pure and blended oils (The data represents the average value).

| **Label** | **Raman features** | | | | | | | | | | | | | | | | | | | | | | | | |
| --- | --- | --- | --- | --- | --- | --- | --- | --- | --- | --- | --- | --- | --- | --- | --- | --- | --- | --- | --- | --- | --- | --- | --- | --- | --- |
|  | **Including peak intensity (I)** | | | | | | | | | | **Peak area (A)** | | | | | **Peak area ratio (R)** | | | | | **Full width at half maximum (F)** | | | | |
|  | **I970** | **I1005** | **I1075** | **I1156** | **I1265** | **I1302** | **I1440** | **I1526** | **I1658** | **I1746** | **A1156** | **A1265** | **A1440** | **A1526** | **A1658** | **R1156** | **R1265** | **R1440** | **R1526** | **R1658** | **F1156** | **F1265** | **F1440** | **F1526** | **F1658** |
| **OO1** | 0.060 | 0.158 | 0.280 | 0.260 | 0.422 | 0.687 | 1.000 | 0.507 | 0.758 | 0.236 | 1789.4 | 11630.3 | 10086.1 | 3941.7 | 5727.1 | 0.05 | 0.35 | 0.30 | 0.12 | 0.17 | 3.00 | 9.13 | 5.57 | 4.11 | 3.68 |
| **OO2** | 0.117 | 0.286 | 0.316 | 0.323 | 0.485 | 0.761 | 1.000 | 0.689 | 0.758 | 0.304 | 2410.1 | 14394.3 | 10805.1 | 5826.4 | 5882.7 | 0.06 | 0.37 | 0.27 | 0.15 | 0.15 | 3.30 | 9.93 | 5.38 | 4.22 | 3.44 |
| **OO3** | 0.036 | 0.179 | 0.263 | 0.405 | 0.394 | 0.682 | 1.000 | 0.716 | 0.729 | 0.229 | 2664.9 | 11750.4 | 9785.7 | 5565.4 | 5551.9 | 0.08 | 0.33 | 0.28 | 0.16 | 0.16 | 3.48 | 7.16 | 5.32 | 4.21 | 3.51 |
| **OO4** | 0.134 | 0.347 | 0.321 | 0.446 | 0.488 | 0.793 | 1.000 | 0.849 | 0.746 | 0.340 | 3116.3 | 14954.8 | 10397.8 | 7177.7 | 5578.6 | 0.08 | 0.36 | 0.25 | 0.18 | 0.14 | 3.22 | 9.61 | 5.27 | 4.21 | 3.28 |
| **OO5** | 0.054 | 0.234 | 0.254 | 0.497 | 0.420 | 0.699 | 1.000 | 0.843 | 0.770 | 0.231 | 2690.1 | 9502.4 | 7974.1 | 5246.3 | 4527.6 | 0.09 | 0.32 | 0.27 | 0.18 | 0.15 | 3.51 | 6.23 | 5.34 | 4.05 | 3.32 |
| **OO6** | 0.048 | 0.216 | 0.305 | 0.389 | 0.456 | 0.705 | 1.000 | 0.723 | 0.743 | 0.293 | 1965.5 | 9817.6 | 8643.2 | 6425.6 | 5692.4 | 0.06 | 0.34 | 0.28 | 0.12 | 0.15 | 3.27 | 8.24 | 5.17 | 4.19 | 3.29 |
| **OO7** | 0.105 | 0.331 | 0.297 | 0..454 | 0.434 | 0.752 | 1.000 | 0.644 | 0.719 | 0.264 | 2047.2 | 12472.7 | 8876.5 | 5242.7 | 4943.1 | 0.05 | 0.35 | 0.28 | 0.17 | 0.15 | 3.42 | 7.47 | 5.68 | 4.17 | 3.36 |
| **OO8** | 0.127 | 0.304 | 0.268 | 0.372 | 0.401 | 0.733 | 1.000 | 0.815 | 0.734 | 0.317 | 3013.6 | 12395.5 | 10764.8 | 5344.7 | 5137.7 | 0.08 | 0.32 | 0.25 | 0.15 | 0.14 | 3.23 | 8.67 | 5.42 | 4.20 | 3.57 |
| **OO9** | 0.067 | 0.193 | 0.289 | 0.326 | 0.465 | 0.717 | 1.000 | 0.656 | 0.789 | 0.245 | 2771.3 | 13578.2 | 9975.3 | 5407.4 | 5676.2 | 0.09 | 0.33 | 0.26 | 0.13 | 0.15 | 3.26 | 8.59 | 5.43 | 4.07 | 3.41 |
| **OO10** | 0.052 | 0.162 | 0.278 | 0.457 | 0.457 | 0.711 | 1.000 | 0.765 | 0.774 | 0.216 | 2873.9 | 13967.7 | 10788.4 | 5337.2 | 5817.5 | 0.06 | 0.33 | 0.26 | 0.14 | 0.14 | 3.31 | 9.04 | 5.10 | 4.18 | 3.53 |
| **SO1** | 0.260 | 0.272 | 0.414 | 0.012 | 0.804 | 0.818 | 1.000 | 0.119 | 1.245 | 0.354 | 0.0 | 10897.5 | 6534.1 | 0.0 | 5979.2 | 0.00 | 0.46 | 0.28 | 0.00 | 0.26 | 0.00 | 12.40 | 5.64 | 0.00 | 3.56 |
| **SO2** | 0.342 | 0.403 | 0.483 | 0.027 | 0.902 | 0.922 | 1.000 | 0.183 | 1.292 | 0.420 | 0.0 | 12451.3 | 6556.2 | 0.0 | 6053.5 | 0.00 | 0.49 | 0.26 | 0.00 | 0.24 | 0.00 | 12.80 | 5.55 | 0.00 | 3.45 |
| **SO3** | 0.223 | 0.234 | 0.399 | 0.015 | 0.768 | 0.795 | 1.000 | 0.110 | 1.255 | 0.323 | 0.0 | 9685.6 | 6248.0 | 0.0 | 5705.3 | 0.00 | 0.45 | 0.29 | 0.00 | 0.26 | 0.00 | 12.25 | 5.62 | 0.00 | 3.58 |
| **SO4** | 0.284 | 0.315 | 0.434 | 0.025 | 0.897 | 0.867 | 1.000 | 0.162 | 1.353 | 0.390 | 0.0 | 12191.0 | 6898.8 | 0.0 | 6591.0 | 0.00 | 0.47 | 0.27 | 0.00 | 0.26 | 0.00 | 12.66 | 5.58 | 0.00 | 3.45 |
| **SO5** | 0.234 | 0.257 | 0.425 | 0.016 | 0.815 | 0.823 | 1.000 | 0.156 | 1.277 | 0.334 | 0.0 | 11573.4 | 6314.3 | 0.0 | 6243.3 | 0.00 | 0.48 | 0.25 | 0.00 | 0.25 | 0.00 | 12.57 | 5.59 | 0.00 | 3.51 |
| **SO6** | 0.335 | 0.351 | 0.463 | 0.023 | 0.845 | 0.872 | 1.000 | 0.137 | 1.249 | 0.364 | 0.0 | 12267.4 | 6426.1 | 0.0 | 6367.5 | 0.00 | 0.44 | 0.26 | 0.00 | 0.24 | 0.00 | 12.46 | 5.60 | 0.00 | 3.57 |
| **SO7** | 0.275 | 0.327 | 0.398 | 0.022 | 0.872 | 0.904 | 1.000 | 0.175 | 1.254 | 0.417 | 0.0 | 9942.7 | 6720.7 | 0.0 | 5847.2 | 0.00 | 0.47 | 0.28 | 0.00 | 0.25 | 0.00 | 12.30 | 5.61 | 0.00 | 3.55 |
| **SO8** | 0.306 | 0.286 | 0.443 | 0.024 | 0.854 | 0.853 | 1.000 | 0.146 | 1.312 | 0.382 | 0.0 | 10763.7 | 6643.5 | 0.0 | 5776.4 | 0.00 | 0.45 | 0.27 | 0.00 | 0.24 | 0.00 | 12.75 | 5.57 | 0.00 | 3.44 |
| **SO9** | 0.273 | 0.343 | 0.452 | 0.017 | 0.863 | 0.834 | 1.000 | 0.122 | 1.305 | 0.378 | 0.0 | 11924.2 | 6828.2 | 0.0 | 6172.9 | 0.00 | 0.44 | 0.25 | 0.00 | 0.26 | 0.00 | 12.59 | 5.56 | 0.00 | 3.52 |
| **SO10** | 0.242 | 0.273 | 0.421 | 0.019 | 0.812 | 0.821 | 1.000 | 0.131 | 1.316 | 0.361 | 0.0 | 11365.8 | 6422.3 | 0.0 | 6085.6 | 0.00 | 0.45 | 0.28 | 0.00 | 0.24 | 0.00 | 12.42 | 5.58 | 0.00 | 3.47 |
| **RO1** | 0.683 | 1.158 | 0.687 | 0.670 | 0.998 | 1.242 | 1.000 | 1.717 | 1.032 | 0.789 | 1495.6 | 7239.3 | 2865.2 | 5296.7 | 2473.3 | 0.08 | 0.37 | 0.15 | 0.27 | 0.13 | 2.84 | 11.68 | 4.14 | 4.69 | 3.03 |
| **RO2** | 0.458 | 0.750 | 0.613 | 0.177 | 0.856 | 1.027 | 1.000 | 0.774 | 1.082 | 0.595 | 504.6 | 5216.3 | 2518.8 | 2289.2 | 2084.7 | 0.04 | 0.41 | 0.20 | 0.18 | 0.17 | 1.88 | 11.28 | 4.69 | 5.12 | 3.16 |
| **RO3** | 0.319 | 0.491 | 0.479 | 0.182 | 0.793 | 0.943 | 1.000 | 0.568 | 1.082 | 0.503 | 398.0 | 5238.0 | 2825.9 | 1566.1 | 2389.9 | 0.03 | 0.42 | 0.23 | 0.13 | 0.19 | 2.92 | 12.16 | 5.50 | 5.11 | 3.53 |
| **RO4** | 0.520 | 0.896 | 0.598 | 0.535 | 0.878 | 1.054 | 1.000 | 1.323 | 1.046 | 0.639 | 1254.4 | 6204.0 | 2911.8 | 3736.8 | 2322.7 | 0.08 | 0.38 | 0.18 | 0.23 | 0.14 | 3.27 | 11.81 | 4.61 | 4.51 | 3.08 |
| **RO5** | 0.506 | 0.613 | 0.535 | 0.246 | 0.824 | 1.005 | 1.000 | 0.846 | 1.054 | 0.624 | 495.5 | 5562.1 | 2798.4 | 2893.3 | 2567.2 | 0.05 | 0.40 | 0.17 | 0.22 | 0.15 | 2.56 | 11.95 | 4.27 | 4.87 | 3.25 |
| **RO6** | 0.499 | 0.953 | 0.597 | 0.457 | 0.862 | 1.098 | 1.000 | 1.254 | 1.073 | 0.568 | 846.5 | 5623.8 | 2624.7 | 4375.6 | 2465.6 | 0.07 | 0.43 | 0.21 | 0.24 | 0.18 | 2.37 | 11.27 | 4.37 | 4.93 | 3.22 |
| **RO7** | 0.613 | 0.802 | 0.649 | 0.563 | 0.945 | 1.176 | 1.000 | 1.625 | 1.062 | 0.647 | 1098.1 | 6142.2 | 2852.3 | 3921.9 | 2561.4 | 0.06 | 0.40 | 0.21 | 0.19 | 0.12 | 2.62 | 11.57 | 4.52 | 5.07 | 3.34 |
| **RO8** | 0.387 | 0.942 | 0.524 | 0.460 | 0.912 | 0.974 | 1.000 | 0.955 | 1.055 | 0.686 | 1164.7 | 6821.6 | 2763.6 | 2176.5 | 2156.1 | 0.06 | 0.35 | 0.18 | 0.26 | 0.16 | 2.86 | 11.75 | 5.15 | 4.75 | 3.22 |
| **RO9** | 0.412 | 0.796 | 0.576 | 0.349 | 0.943 | 0.957 | 1.000 | 0.642 | 1.074 | 0.643 | 716.3 | 6432.4 | 2752.4 | 2236.7 | 2093.5 | 0.08 | 0.36 | 0.20 | 0.17 | 0.15 | 2.89 | 12.08 | 5.31 | 4.62 | 3.11 |
| **RO10** | 0.554 | 0.841 | 0.678 | 0.274 | 0.802 | 1.195 | 1.000 | 1.253 | 1.052 | 0.628 | 1157.2 | 5264.4 | 2891.1 | 3729.4 | 2061.2 | 0.05 | 0.38 | 0.16 | 0.16 | 0.13 | 2.98 | 11.74 | 4.73 | 4.82 | 3.15 |
| **CO1** | 0.136 | 0.082 | 0.324 | 0.008 | 0.633 | 0.641 | 1.000 | 0.033 | 1.189 | 0.195 | 0.0 | 7589.1 | 6709.0 | 0.0 | 5224.8 | 0.00 | 0.39 | 0.34 | 0.00 | 0.27 | 0.00 | 11.64 | 5.82 | 0.00 | 3.60 |
| **CO2** | 0.160 | 0.129 | 0.345 | 0.016 | 0.677 | 0.684 | 1.000 | 0.046 | 1.178 | 0.253 | 0.0 | 7950.2 | 6426.7 | 0.0 | 5362.7 | 0.00 | 0.40 | 0.33 | 0.00 | 0.27 | 0.00 | 11.84 | 5.78 | 0.00 | 3.69 |
| **CO3** | 0.185 | 0.174 | 0.366 | 0.010 | 0.721 | 0.717 | 1.000 | 0.072 | 1.205 | 0.292 | 0.0 | 9500.3 | 7006.0 | 0.0 | 5720.0 | 0.00 | 0.43 | 0.32 | 0.00 | 0.26 | 0.00 | 11.96 | 5.66 | 0.00 | 3.53 |
| **CO4** | 0.161 | 0.126 | 0.354 | 0.010 | 0.686 | 0.704 | 1.000 | 0.065 | 1.247 | 0.236 | 0.0 | 9128.0 | 7278.0 | 0.0 | 6002.5 | 0.00 | 0.41 | 0.32 | 0.00 | 0.27 | 0.00 | 11.86 | 5.78 | 0.00 | 3.59 |
| **CO5** | 0.157 | 0.145 | 0.368 | 0.009 | 0.681 | 0.654 | 1.000 | 0.061 | 1.162 | 0.261 | 0.0 | 8612.7 | 7161.5 | 0.0 | 5467.2 | 0.00 | 0.41 | 0.33 | 0.00 | 0.27 | 0.00 | 11.72 | 5.72 | 0.00 | 3.54 |
| **CO6** | 0.176 | 0.132 | 0.338 | 0.011 | 0.642 | 0.722 | 1.000 | 0.055 | 1.254 | 0.226 | 0.0 | 8225.4 | 6893.8 | 0.0 | 5696.1 | 0.00 | 0.41 | 0.32 | 0.00 | 0.26 | 0.00 | 11.76 | 5.76 | 0.00 | 3.62 |
| **CO7** | 0.145 | 0.167 | 0.382 | 0.012 | 0.664 | 0.671 | 1.000 | 0.054 | 1.192 | 0.217 | 0.0 | 8739.5 | 6844.3 | 0.0 | 5613.3 | 0.00 | 0.38 | 0.31 | 0.00 | 0.26 | 0.00 | 11.67 | 5.73 | 0.00 | 3.68 |
| **CO8** | 0.172 | 0.113 | 0.338 | 0.015 | 0.715 | 0.689 | 1.000 | 0.055 | 1.236 | 0.245 | 0.0 | 9073.5 | 7254.6 | 0.0 | 5848.4 | 0.00 | 0.38 | 0.32 | 0.00 | 0.26 | 0.00 | 11.88 | 5.79 | 0.00 | 3.67 |
| **CO9** | 0.164 | 0.122 | 0.326 | 0.012 | 0.692 | 0.696 | 1.000 | 0.052 | 1.235 | 0.259 | 0.0 | 8443.6 | 6582.1 | 0.0 | 5352.4 | 0.00 | 0.42 | 0.33 | 0.00 | 0.27 | 0.00 | 11.92 | 5.74 | 0.00 | 3.66 |
| **CO10** | 0.155 | 0.093 | 0.329 | 0.009 | 0.682 | 0.693 | 1.000 | 0.051 | 1.154 | 0.256 | 0.0 | 8156.2 | 6393.2 | 0.0 | 5487.2 | 0.00 | 0.39 | 0.31 | 0.00 | 0.25 | 0.00 | 11.97 | 5.81 | 0.00 | 3.52 |
| **OS0** | 0.323 | 0.397 | 0.489 | 0.032 | 0.918 | 0.908 | 1.000 | 0.153 | 1.231 | 0.409 | 146.7 | 12080.7 | 6409.6 | 813.1 | 5733.5 | 0.01 | 0.48 | 0.26 | 0.03 | 0.23 | 0.77 | 12.79 | 5.60 | 4.63 | 3.59 |
| **OS10** | 0.228 | 0.245 | 0.386 | 0.037 | 0.751 | 0.776 | 1.000 | 0.159 | 1.179 | 0.332 | 155.7 | 10105.3 | 6467.2 | 826.6 | 5492.6 | 0.01 | 0.44 | 0.28 | 0.04 | 0.24 | 1.12 | 12.29 | 5.67 | 4.79 | 3.60 |
| **OS20** | 0.196 | 0.231 | 0.378 | 0.046 | 0.720 | 0.783 | 1.000 | 0.192 | 1.142 | 0.319 | 249.2 | 9702.6 | 6660.1 | 1079.4 | 5400.5 | 0.01 | 0.42 | 0.29 | 0.05 | 0.23 | 1.74 | 11.90 | 5.73 | 4.89 | 3.56 |
| **OS30** | 0.167 | 0.203 | 0.346 | 0.044 | 0.650 | 0.733 | 1.000 | 0.220 | 1.112 | 0.282 | 313.0 | 9178.3 | 6436.3 | 1326.2 | 5220.4 | 0.01 | 0.41 | 0.29 | 0.06 | 0.23 | 1.54 | 11.62 | 5.42 | 4.42 | 3.52 |
| **OS40** | 0.134 | 0.168 | 0.315 | 0.069 | 0.600 | 0.705 | 1.000 | 0.251 | 1.102 | 0.262 | 487.9 | 9049.4 | 6760.1 | 1535.9 | 5199.0 | 0.02 | 0.39 | 0.29 | 0.07 | 0.23 | 1.99 | 11.11 | 5.51 | 3.89 | 3.44 |
| **OS50** | 0.110 | 0.146 | 0.297 | 0.118 | 0.544 | 0.689 | 1.000 | 0.290 | 1.031 | 0.236 | 594.6 | 8271.9 | 6744.8 | 1652.9 | 4761.9 | 0.03 | 0.38 | 0.31 | 0.08 | 0.22 | 1.80 | 10.54 | 5.43 | 3.84 | 3.41 |
| **OS60** | 0.090 | 0.142 | 0.294 | 0.139 | 0.523 | 0.666 | 1.000 | 0.327 | 0.986 | 0.248 | 680.2 | 7991.1 | 6450.6 | 1801.2 | 4583.0 | 0.03 | 0.37 | 0.30 | 0.08 | 0.21 | 2.13 | 10.61 | 5.39 | 3.80 | 3.43 |
| **OS70** | 0.082 | 0.132 | 0.275 | 0.169 | 0.483 | 0.658 | 1.000 | 0.343 | 0.949 | 0.230 | 847.5 | 8076.9 | 6477.1 | 1884.5 | 4503.1 | 0.04 | 0.37 | 0.30 | 0.09 | 0.21 | 3.03 | 10.39 | 5.35 | 3.88 | 3.37 |
| **OS80** | 0.080 | 0.150 | 0.287 | 0.214 | 0.470 | 0.674 | 1.000 | 0.408 | 0.894 | 0.224 | 919.2 | 8114.0 | 6774.1 | 2338.1 | 4367.8 | 0.04 | 0.36 | 0.30 | 0.10 | 0.19 | 2.72 | 10.09 | 5.37 | 4.06 | 3.37 |
| **OS90** | 0.063 | 0.121 | 0.276 | 0.240 | 0.446 | 0.670 | 1.000 | 0.450 | 0.837 | 0.230 | 992.4 | 7888.0 | 6335.0 | 2395.4 | 4034.9 | 0.05 | 0.36 | 0.30 | 0.11 | 0.18 | 2.93 | 9.46 | 5.35 | 3.98 | 3.33 |
| **OS100** | 0.056 | 0.141 | 0.270 | 0.288 | 0.411 | 0.660 | 1.000 | 0.487 | 0.807 | 0.227 | 1098.9 | 7419.6 | 6517.5 | 2444.7 | 3698.5 | 0.05 | 0.35 | 0.31 | 0.12 | 0.17 | 3.14 | 8.82 | 5.33 | 3.89 | 3.30 |
| **OR0** | 0.808 | 1.337 | 0.790 | 0.681 | 1.122 | 1.326 | 1.000 | 1.788 | 1.033 | 0.881 | 1601.2 | 8303.0 | 2748.5 | 5310.5 | 2484.4 | 0.08 | 0.41 | 0.13 | 0.26 | 0.12 | 3.18 | 12.30 | 4.15 | 4.80 | 2.99 |
| **OR10** | 0.639 | 1.095 | 0.660 | 0.583 | 1.008 | 1.258 | 1.000 | 1.535 | 1.004 | 0.796 | 1378.3 | 7661.2 | 2932.8 | 4755.2 | 2390.5 | 0.07 | 0.40 | 0.15 | 0.25 | 0.13 | 2.84 | 11.56 | 4.39 | 4.68 | 3.05 |
| **OR20** | 0.498 | 0.915 | 0.598 | 0.580 | 0.893 | 1.116 | 1.000 | 1.410 | 0.987 | 0.682 | 1248.3 | 6627.7 | 2820.9 | 3940.5 | 2223.6 | 0.07 | 0.39 | 0.17 | 0.23 | 0.13 | 2.83 | 11.73 | 4.52 | 4.44 | 3.02 |
| **OR30** | 0.435 | 0.794 | 0.534 | 0.533 | 0.807 | 1.012 | 1.000 | 1.261 | 0.921 | 0.628 | 1319.5 | 6813.2 | 3197.0 | 4106.0 | 2408.0 | 0.07 | 0.38 | 0.18 | 0.23 | 0.14 | 2.78 | 11.44 | 4.73 | 4.40 | 3.11 |
| **OR40** | 0.399 | 0.754 | 0.522 | 0.501 | 0.763 | 0.977 | 1.000 | 1.199 | 0.941 | 0.589 | 1140.1 | 5814.2 | 2946.4 | 3576.0 | 2210.4 | 0.07 | 0.37 | 0.19 | 0.23 | 0.14 | 2.95 | 11.32 | 4.77 | 4.43 | 3.14 |
| **OR50** | 0.278 | 0.559 | 0.428 | 0.449 | 0.657 | 0.884 | 1.000 | 1.009 | 0.898 | 0.479 | 1071.1 | 5508.7 | 3089.8 | 3207.8 | 2154.4 | 0.07 | 0.37 | 0.21 | 0.21 | 0.14 | 2.89 | 10.29 | 4.76 | 4.26 | 3.16 |
| **OR60** | 0.251 | 0.469 | 0.403 | 0.438 | 0.622 | 0.848 | 1.000 | 0.909 | 0.910 | 0.429 | 1020.0 | 5226.2 | 3130.4 | 2731.7 | 2229.7 | 0.07 | 0.36 | 0.22 | 0.19 | 0.16 | 2.88 | 10.26 | 4.89 | 4.24 | 3.17 |
| **OR70** | 0.204 | 0.435 | 0.388 | 0.380 | 0.599 | 0.859 | 1.000 | 0.834 | 0.894 | 0.397 | 839.0 | 5093.9 | 3128.1 | 2408.9 | 2115.7 | 0.06 | 0.37 | 0.23 | 0.18 | 0.16 | 2.98 | 9.87 | 5.09 | 4.16 | 3.24 |
| **OR80** | 0.150 | 0.303 | 0.332 | 0.367 | 0.506 | 0.746 | 1.000 | 0.690 | 0.855 | 0.315 | 819.4 | 4626.9 | 3320.2 | 2096.4 | 2137.2 | 0.06 | 0.36 | 0.26 | 0.16 | 0.16 | 2.71 | 8.49 | 5.11 | 4.14 | 3.24 |
| **OR90** | 0.103 | 0.245 | 0.305 | 0.330 | 0.488 | 0.710 | 1.000 | 0.620 | 0.859 | 0.301 | 732.2 | 4227.3 | 3235.6 | 1670.0 | 1981.7 | 0.06 | 0.36 | 0.27 | 0.14 | 0.17 | 2.94 | 8.99 | 5.25 | 3.99 | 3.26 |
| **OR100** | 0.066 | 0.179 | 0.270 | 0.304 | 0.418 | 0.661 | 1.000 | 0.505 | 0.834 | 0.242 | 566.0 | 3593.1 | 3157.3 | 1233.1 | 1825.0 | 0.05 | 0.35 | 0.30 | 0.12 | 0.18 | 2.84 | 8.25 | 5.36 | 3.78 | 3.29 |
| **OC0** | 0.145 | 0.095 | 0.331 | 0.004 | 0.657 | 0.671 | 1.000 | 0.054 | 1.194 | 0.243 | 29.7 | 8839.3 | 7233.7 | 389.7 | 5962.7 | 0.00 | 0.39 | 0.32 | 0.02 | 0.27 | 0.52 | 11.77 | 5.79 | 1.76 | 3.63 |
| **OC10** | 0.129 | 0.091 | 0.310 | 0.020 | 0.614 | 0.650 | 1.000 | 0.097 | 1.148 | 0.236 | 63.7 | 8505.7 | 7045.7 | 526.3 | 5788.8 | 0.00 | 0.39 | 0.32 | 0.02 | 0.26 | 0.73 | 11.77 | 5.68 | 2.37 | 3.54 |
| **OC20** | 0.129 | 0.117 | 0.306 | 0.041 | 0.597 | 0.683 | 1.000 | 0.145 | 1.088 | 0.238 | 179.4 | 8690.3 | 6844.0 | 641.1 | 5280.6 | 0.01 | 0.40 | 0.32 | 0.03 | 0.24 | 1.64 | 11.44 | 5.68 | 3.75 | 3.55 |
| **OC30** | 0.118 | 0.124 | 0.310 | 0.096 | 0.573 | 0.659 | 1.000 | 0.197 | 1.080 | 0.246 | 362.8 | 7003.8 | 5897.2 | 769.2 | 4508.8 | 0.02 | 0.38 | 0.32 | 0.04 | 0.24 | 2.27 | 11.28 | 5.74 | 4.59 | 3.46 |
| **OC40** | 0.112 | 0.121 | 0.289 | 0.092 | 0.548 | 0.650 | 1.000 | 0.233 | 1.015 | 0.235 | 458.1 | 7432.6 | 6320.9 | 1127.1 | 4555.3 | 0.02 | 0.37 | 0.32 | 0.06 | 0.23 | 2.56 | 10.97 | 5.69 | 4.48 | 3.48 |
| **OC50** | 0.084 | 0.095 | 0.274 | 0.124 | 0.489 | 0.622 | 1.000 | 0.266 | 0.991 | 0.204 | 644.3 | 7780.8 | 6940.4 | 1456.7 | 4888.7 | 0.03 | 0.36 | 0.32 | 0.07 | 0.23 | 2.49 | 10.53 | 5.43 | 3.65 | 3.40 |
| **OC60** | 0.073 | 0.102 | 0.263 | 0.152 | 0.477 | 0.646 | 1.000 | 0.307 | 0.973 | 0.214 | 651.4 | 7344.1 | 6469.5 | 1563.4 | 4373.6 | 0.03 | 0.36 | 0.32 | 0.08 | 0.21 | 2.30 | 10.31 | 5.39 | 3.56 | 3.37 |
| **OC70** | 0.071 | 0.110 | 0.277 | 0.182 | 0.464 | 0.665 | 1.000 | 0.350 | 0.941 | 0.214 | 746.9 | 7043.0 | 6273.2 | 1710.0 | 4089.1 | 0.04 | 0.35 | 0.32 | 0.09 | 0.21 | 2.90 | 9.78 | 5.50 | 3.84 | 3.38 |
| **OC80** | 0.079 | 0.128 | 0.270 | 0.212 | 0.461 | 0.658 | 1.000 | 0.388 | 0.908 | 0.225 | 813.4 | 7204.5 | 6474.7 | 2072.3 | 4101.9 | 0.04 | 0.35 | 0.31 | 0.10 | 0.20 | 2.77 | 9.68 | 5.40 | 3.92 | 3.32 |
| **OC90** | 0.061 | 0.147 | 0.275 | 0.247 | 0.434 | 0.668 | 1.000 | 0.450 | 0.869 | 0.218 | 941.5 | 7450.4 | 6473.5 | 2284.4 | 3836.5 | 0.04 | 0.36 | 0.31 | 0.11 | 0.18 | 3.01 | 9.12 | 5.44 | 3.81 | 3.32 |
| **OC100** | 0.048 | 0.152 | 0.276 | 0.289 | 0.412 | 0.681 | 1.000 | 0.504 | 0.838 | 0.239 | 1130.4 | 7483.3 | 6418.4 | 2459.1 | 3683.2 | 0.05 | 0.35 | 0.30 | 0.12 | 0.17 | 3.04 | 7.83 | 5.37 | 3.85 | 3.27 |
| **OSR1** | 0.641 | 1.064 | 0.628 | 0.617 | 0.961 | 1.136 | 1.000 | 1.635 | 0.983 | 0.733 | 1583.1 | 7211.9 | 3015.6 | 5154.5 | 2456.7 | 0.08 | 0.37 | 0.16 | 0.26 | 0.13 | 3.07 | 11.76 | 4.42 | 4.59 | 3.19 |
| **OSR2** | 0.530 | 0.863 | 0.577 | 0.388 | 0.923 | 1.033 | 1.000 | 1.164 | 1.076 | 0.657 | 1131.5 | 7392.0 | 3373.3 | 4208.0 | 2857.4 | 0.06 | 0.39 | 0.18 | 0.22 | 0.15 | 2.73 | 11.93 | 4.64 | 4.75 | 3.21 |
| **OSR3** | 0.384 | 0.625 | 0.493 | 0.246 | 0.819 | 0.921 | 1.000 | 0.821 | 1.165 | 0.487 | 682.2 | 5757.8 | 2906.4 | 2524.6 | 2677.7 | 0.05 | 0.39 | 0.20 | 0.17 | 0.18 | 2.21 | 11.24 | 4.77 | 4.37 | 3.35 |
| **OSR4** | 0.386 | 0.529 | 0.508 | 0.157 | 0.916 | 0.951 | 1.000 | 0.504 | 1.208 | 0.508 | 380.4 | 6846.5 | 3221.2 | 1523.5 | 2988.3 | 0.03 | 0.46 | 0.22 | 0.10 | 0.20 | 2.42 | 12.83 | 5.47 | 5.23 | 3.51 |
| **OSR5** | 0.371 | 0.439 | 0.526 | 0.064 | 0.912 | 0.928 | 1.000 | 0.193 | 1.241 | 0.459 | 127.8 | 6690.6 | 3392.9 | 624.6 | 3193.5 | 0.01 | 0.48 | 0.24 | 0.04 | 0.23 | 0.78 | 12.97 | 5.62 | 4.19 | 3.52 |
| **OSR6** | 0.570 | 1.048 | 0.655 | 0.638 | 0.950 | 1.137 | 1.000 | 1.577 | 1.044 | 0.722 | 1501.3 | 7570.4 | 3436.0 | 5256.1 | 2555.2 | 0.08 | 0.37 | 0.16 | 0.26 | 0.13 | 2.94 | 11.64 | 4.38 | 4.60 | 3.01 |
| **OSR7** | 0.449 | 0.767 | 0.542 | 0.463 | 0.864 | 1.007 | 1.000 | 1.184 | 1.136 | 0.562 | 1008.9 | 5866.4 | 2743.2 | 3347.2 | 2373.0 | 0.07 | 0.38 | 0.18 | 0.22 | 0.15 | 2.85 | 10.83 | 4.57 | 4.31 | 3.08 |
| **OSR8** | 0.369 | 0.588 | 0.512 | 0.314 | 0.808 | 0.899 | 1.000 | 0.820 | 1.185 | 0.491 | 766.5 | 5400.1 | 2939.2 | 2530.3 | 2606.7 | 0.05 | 0.38 | 0.21 | 0.18 | 0.18 | 2.60 | 11.50 | 4.92 | 4.66 | 3.20 |
| **OSR9** | 0.253 | 0.296 | 0.403 | 0.044 | 0.765 | 0.800 | 1.000 | 0.181 | 1.236 | 0.363 | 77.7 | 5037.3 | 3058.2 | 515.2 | 2788.5 | 0.01 | 0.44 | 0.27 | 0.04 | 0.24 | 0.78 | 12.60 | 5.48 | 3.22 | 3.43 |
| **OSR10** | 0.517 | 0.905 | 0.600 | 0.639 | 0.903 | 1.106 | 1.000 | 1.443 | 1.103 | 0.659 | 1251.0 | 6450.0 | 2833.0 | 3923.3 | 2316.1 | 0.07 | 0.38 | 0.17 | 0.23 | 0.14 | 2.90 | 11.65 | 4.59 | 4.26 | 2.94 |
| **OSR11** | 0.464 | 0.811 | 0.548 | 0.468 | 0.877 | 1.058 | 1.000 | 1.175 | 1.123 | 0.601 | 932.9 | 5325.7 | 2542.4 | 3125.1 | 2168.6 | 0.07 | 0.38 | 0.18 | 0.22 | 0.15 | 2.70 | 10.90 | 4.60 | 4.44 | 2.97 |
| **OSR12** | 0.341 | 0.555 | 0.467 | 0.273 | 0.748 | 0.896 | 1.000 | 0.754 | 1.089 | 0.463 | 778.2 | 6202.2 | 3273.1 | 2504.0 | 2739.7 | 0.05 | 0.40 | 0.21 | 0.16 | 0.18 | 2.39 | 11.05 | 4.80 | 4.43 | 3.20 |
| **OSR13** | 0.357 | 0.501 | 0.493 | 0.171 | 0.798 | 0.889 | 1.000 | 0.485 | 1.162 | 0.467 | 425.2 | 5828.3 | 3194.1 | 1537.1 | 2686.6 | 0.03 | 0.43 | 0.23 | 0.11 | 0.20 | 2.31 | 12.14 | 5.38 | 5.69 | 3.23 |
| **OSR14** | 0.293 | 0.333 | 0.455 | 0.052 | 0.791 | 0.840 | 1.000 | 0.222 | 1.221 | 0.375 | 97.4 | 5653.6 | 3282.3 | 670.6 | 3003.4 | 0.01 | 0.44 | 0.26 | 0.05 | 0.24 | 1.24 | 12.40 | 5.52 | 5.33 | 3.37 |
| **OSR15** | 0.413 | 0.823 | 0.579 | 0.576 | 0.831 | 1.057 | 1.000 | 1.419 | 1.036 | 0.619 | 1154.6 | 5222.4 | 2569.7 | 3615.2 | 2047.2 | 0.08 | 0.36 | 0.18 | 0.25 | 0.14 | 3.03 | 11.04 | 4.57 | 4.28 | 3.06 |
| **OSR16** | 0.416 | 0.727 | 0.556 | 0.434 | 0.823 | 1.020 | 1.000 | 1.121 | 1.088 | 0.569 | 1011.8 | 6006.2 | 2884.6 | 3527.2 | 2470.4 | 0.06 | 0.38 | 0.18 | 0.22 | 0.16 | 2.86 | 11.11 | 4.52 | 4.36 | 3.05 |
| **OSR17** | 0.352 | 0.559 | 0.523 | 0.365 | 0.805 | 0.935 | 1.000 | 0.855 | 1.176 | 0.517 | 691.2 | 4761.4 | 2521.3 | 2282.3 | 2157.0 | 0.06 | 0.38 | 0.20 | 0.18 | 0.18 | 2.66 | 11.30 | 5.05 | 4.77 | 3.16 |
| **OSR18** | 0.284 | 0.411 | 0.439 | 0.232 | 0.754 | 0.840 | 1.000 | 0.529 | 1.113 | 0.428 | 539.4 | 5440.4 | 3250.6 | 1727.8 | 2742.0 | 0.04 | 0.40 | 0.24 | 0.13 | 0.20 | 2.58 | 11.97 | 5.27 | 5.07 | 3.36 |
| **OSR19** | 0.246 | 0.281 | 0.413 | 0.064 | 0.757 | 0.809 | 1.000 | 0.212 | 1.173 | 0.372 | 115.4 | 6050.6 | 3799.9 | 709.1 | 3276.6 | 0.01 | 0.43 | 0.27 | 0.05 | 0.24 | 1.19 | 12.21 | 5.43 | 5.08 | 3.42 |
| **OSR20** | 0.438 | 0.835 | 0.581 | 0.595 | 0.818 | 1.055 | 1.000 | 1.387 | 1.031 | 0.639 | 1284.2 | 6237.1 | 2971.3 | 3985.1 | 2297.4 | 0.08 | 0.37 | 0.18 | 0.24 | 0.14 | 2.87 | 10.99 | 4.48 | 4.19 | 2.98 |
| **OSR21** | 0.388 | 0.654 | 0.511 | 0.454 | 0.771 | 0.950 | 1.000 | 1.069 | 1.098 | 0.535 | 1059.0 | 6021.6 | 3085.8 | 3383.9 | 2532.5 | 0.07 | 0.37 | 0.19 | 0.21 | 0.16 | 2.91 | 11.15 | 4.74 | 4.32 | 3.06 |
| **OSR22** | 0.297 | 0.470 | 0.431 | 0.288 | 0.704 | 0.839 | 1.000 | 0.752 | 1.130 | 0.413 | 772.3 | 5648.0 | 3431.7 | 2505.3 | 2829.7 | 0.05 | 0.37 | 0.23 | 0.16 | 0.19 | 2.67 | 11.01 | 4.84 | 4.01 | 3.09 |
| **OSR23** | 0.249 | 0.357 | 0.402 | 0.179 | 0.690 | 0.787 | 1.000 | 0.498 | 1.102 | 0.386 | 572.4 | 5957.5 | 3738.2 | 1920.1 | 3244.1 | 0.04 | 0.39 | 0.24 | 0.12 | 0.21 | 2.50 | 11.31 | 5.14 | 4.52 | 3.35 |
| **OSR24** | 0.214 | 0.234 | 0.378 | 0.072 | 0.691 | 0.751 | 1.000 | 0.226 | 1.121 | 0.321 | 133.8 | 4984.8 | 3562.7 | 784.3 | 2822.3 | 0.01 | 0.41 | 0.29 | 0.06 | 0.23 | 1.66 | 11.85 | 5.54 | 5.11 | 3.42 |
| **OSR25** | 0.368 | 0.701 | 0.502 | 0.562 | 0.768 | 0.967 | 1.000 | 1.259 | 1.047 | 0.557 | 1364.1 | 6738.7 | 3342.2 | 4267.2 | 2784.7 | 0.07 | 0.36 | 0.18 | 0.23 | 0.15 | 2.80 | 11.09 | 4.61 | 4.34 | 2.93 |
| **OSR26** | 0.353 | 0.606 | 0.488 | 0.412 | 0.738 | 0.898 | 1.000 | 0.986 | 1.068 | 0.490 | 1035.7 | 6248.8 | 3330.3 | 3400.8 | 2803.8 | 0.06 | 0.37 | 0.20 | 0.20 | 0.17 | 2.80 | 11.15 | 4.76 | 4.21 | 3.10 |
| **OSR27** | 0.273 | 0.470 | 0.449 | 0.326 | 0.701 | 0.846 | 1.000 | 0.758 | 1.089 | 0.435 | 889.6 | 6088.9 | 3692.8 | 2602.5 | 3019.5 | 0.05 | 0.37 | 0.23 | 0.16 | 0.19 | 2.70 | 11.00 | 4.91 | 3.96 | 3.23 |
| **OSR28** | 0.193 | 0.296 | 0.371 | 0.190 | 0.659 | 0.758 | 1.000 | 0.457 | 1.143 | 0.340 | 614.4 | 5588.8 | 3889.1 | 1720.0 | 3431.8 | 0.04 | 0.37 | 0.26 | 0.11 | 0.23 | 2.58 | 10.42 | 5.20 | 4.17 | 3.31 |
| **OSR29** | 0.200 | 0.213 | 0.356 | 0.080 | 0.679 | 0.741 | 1.000 | 0.221 | 1.130 | 0.319 | 187.4 | 6119.4 | 4119.7 | 768.7 | 3573.6 | 0.01 | 0.41 | 0.28 | 0.05 | 0.24 | 1.81 | 11.92 | 5.50 | 4.38 | 3.56 |
| **OSR30** | 0.419 | 0.773 | 0.550 | 0.559 | 0.802 | 1.023 | 1.000 | 1.261 | 1.020 | 0.599 | 1387.5 | 7480.2 | 3575.1 | 4521.7 | 2839.1 | 0.07 | 0.38 | 0.18 | 0.23 | 0.15 | 2.69 | 11.16 | 4.46 | 4.18 | 3.07 |
| **OSR31** | 0.342 | 0.616 | 0.514 | 0.405 | 0.721 | 0.892 | 1.000 | 0.984 | 1.034 | 0.502 | 1070.8 | 6387.8 | 3481.5 | 3499.3 | 2874.5 | 0.06 | 0.37 | 0.20 | 0.20 | 0.17 | 2.83 | 11.14 | 4.70 | 4.17 | 3.15 |
| **OSR32** | 0.325 | 0.545 | 0.480 | 0.280 | 0.719 | 0.886 | 1.000 | 0.761 | 1.104 | 0.456 | 776.0 | 5918.8 | 3352.1 | 2547.5 | 2882.2 | 0.05 | 0.38 | 0.22 | 0.16 | 0.19 | 2.58 | 10.96 | 4.81 | 4.15 | 3.16 |
| **OSR33** | 0.222 | 0.346 | 0.398 | 0.192 | 0.664 | 0.797 | 1.000 | 0.515 | 1.113 | 0.380 | 610.8 | 5888.3 | 3814.6 | 2153.1 | 2977.3 | 0.04 | 0.38 | 0.25 | 0.14 | 0.19 | 2.37 | 11.30 | 5.06 | 4.31 | 3.23 |
| **OSR34** | 0.194 | 0.215 | 0.353 | 0.097 | 0.655 | 0.737 | 1.000 | 0.253 | 1.132 | 0.308 | 284.4 | 5813.1 | 4023.2 | 920.2 | 3369.0 | 0.02 | 0.40 | 0.28 | 0.06 | 0.23 | 2.16 | 11.67 | 5.53 | 4.84 | 3.42 |
| **OSR35** | 0.186 | 0.190 | 0.351 | 0.092 | 0.678 | 0.722 | 1.000 | 0.239 | 1.137 | 0.301 | 301.4 | 5825.5 | 4049.3 | 703.9 | 3323.7 | 0.02 | 0.41 | 0.29 | 0.05 | 0.23 | 2.07 | 11.90 | 5.85 | 4.57 | 3.40 |
| **OSC1** | 0.146 | 0.134 | 0.362 | 0.018 | 0.664 | 0.685 | 1.000 | 0.067 | 1.113 | 0.235 | 55.1 | 7093.1 | 5738.9 | 348.3 | 4556.0 | 0.00 | 0.40 | 0.32 | 0.02 | 0.26 | 0.57 | 12.13 | 6.11 | 1.59 | 4.11 |
| **OSC2** | 0.187 | 0.209 | 0.397 | 0.025 | 0.730 | 0.740 | 1.000 | 0.093 | 1.127 | 0.286 | 78.4 | 8392.1 | 5669.6 | 496.7 | 4823.8 | 0.00 | 0.43 | 0.29 | 0.03 | 0.25 | 0.70 | 12.28 | 5.83 | 1.79 | 3.94 |
| **OSC3** | 0.229 | 0.259 | 0.421 | 0.027 | 0.788 | 0.798 | 1.000 | 0.100 | 1.176 | 0.314 | 89.8 | 8651.3 | 5523.3 | 482.7 | 4705.5 | 0.00 | 0.44 | 0.28 | 0.02 | 0.24 | 0.63 | 12.42 | 5.83 | 2.50 | 3.78 |
| **OSC4** | 0.339 | 0.416 | 0.495 | 0.037 | 0.882 | 0.912 | 1.000 | 0.174 | 1.179 | 0.405 | 122.4 | 10303.9 | 5340.6 | 988.3 | 4728.9 | 0.01 | 0.48 | 0.25 | 0.05 | 0.22 | 0.73 | 12.89 | 5.52 | 5.08 | 3.54 |
| **OSC5** | 0.343 | 0.411 | 0.482 | 0.035 | 0.920 | 0.941 | 1.000 | 0.191 | 1.228 | 0.418 | 147.2 | 11446.8 | 5797.7 | 1197.8 | 5294.2 | 0.01 | 0.48 | 0.24 | 0.05 | 0.22 | 0.63 | 12.92 | 5.63 | 5.97 | 3.52 |
| **OSC6** | 0.135 | 0.103 | 0.322 | 0.022 | 0.642 | 0.684 | 1.000 | 0.077 | 1.129 | 0.239 | 78.3 | 8315.2 | 6461.9 | 462.6 | 5331.4 | 0.00 | 0.40 | 0.31 | 0.02 | 0.26 | 0.76 | 11.92 | 5.74 | 2.07 | 3.69 |
| **OSC7** | 0.187 | 0.188 | 0.371 | 0.019 | 0.697 | 0.728 | 1.000 | 0.098 | 1.179 | 0.289 | 76.0 | 8472.5 | 5824.8 | 489.1 | 5067.4 | 0.00 | 0.42 | 0.29 | 0.02 | 0.26 | 0.68 | 12.09 | 5.58 | 2.51 | 3.62 |
| **OSC8** | 0.187 | 0.205 | 0.382 | 0.035 | 0.722 | 0.755 | 1.000 | 0.114 | 1.204 | 0.301 | 77.9 | 7361.3 | 5234.4 | 518.5 | 4336.7 | 0.00 | 0.42 | 0.30 | 0.03 | 0.25 | 0.99 | 12.21 | 5.63 | 2.67 | 3.53 |
| **OSC9** | 0.246 | 0.269 | 0.403 | 0.031 | 0.765 | 0.800 | 1.000 | 0.148 | 1.200 | 0.339 | 111.7 | 10550.6 | 6549.0 | 815.4 | 5803.9 | 0.00 | 0.44 | 0.28 | 0.03 | 0.24 | 0.71 | 12.33 | 5.56 | 4.33 | 3.49 |
| **OSC10** | 0.244 | 0.274 | 0.431 | 0.025 | 0.824 | 0.825 | 1.000 | 0.151 | 1.240 | 0.357 | 90.9 | 9534.2 | 5704.6 | 781.3 | 5052.6 | 0.00 | 0.45 | 0.27 | 0.04 | 0.24 | 0.71 | 12.43 | 5.60 | 4.76 | 3.46 |
| **OSC11** | 0.149 | 0.121 | 0.328 | 0.022 | 0.615 | 0.651 | 1.000 | 0.094 | 1.130 | 0.227 | 78.2 | 6490.3 | 5496.5 | 399.8 | 4409.8 | 0.00 | 0.38 | 0.33 | 0.02 | 0.26 | 0.82 | 11.37 | 5.73 | 2.22 | 3.59 |
| **OSC12** | 0.151 | 0.117 | 0.331 | 0.031 | 0.633 | 0.671 | 1.000 | 0.090 | 1.148 | 0.259 | 108.3 | 7490.4 | 6110.0 | 499.7 | 5032.1 | 0.01 | 0.39 | 0.32 | 0.03 | 0.26 | 1.01 | 11.64 | 5.62 | 2.56 | 3.56 |
| **OSC13** | 0.187 | 0.174 | 0.352 | 0.028 | 0.688 | 0.729 | 1.000 | 0.116 | 1.187 | 0.292 | 97.6 | 9080.6 | 6547.4 | 631.3 | 5608.4 | 0.00 | 0.41 | 0.30 | 0.03 | 0.26 | 0.94 | 11.87 | 5.61 | 3.15 | 3.49 |
| **OSC14** | 0.233 | 0.254 | 0.413 | 0.036 | 0.764 | 0.814 | 1.000 | 0.155 | 1.214 | 0.347 | 96.5 | 9084.2 | 5850.8 | 915.8 | 5135.6 | 0.00 | 0.43 | 0.28 | 0.04 | 0.24 | 1.05 | 12.27 | 5.50 | 4.52 | 3.46 |
| **OSC15** | 0.248 | 0.273 | 0.420 | 0.040 | 0.778 | 0.823 | 1.000 | 0.169 | 1.227 | 0.370 | 129.3 | 9164.2 | 5786.4 | 914.8 | 5101.9 | 0.01 | 0.43 | 0.28 | 0.04 | 0.24 | 1.10 | 12.11 | 5.46 | 4.61 | 3.42 |
| **OSC16** | 0.134 | 0.133 | 0.322 | 0.044 | 0.618 | 0.665 | 1.000 | 0.123 | 1.129 | 0.265 | 131.4 | 7088.2 | 5644.4 | 512.8 | 4430.1 | 0.01 | 0.40 | 0.32 | 0.03 | 0.25 | 1.15 | 11.76 | 5.60 | 2.97 | 3.52 |
| **OSC17** | 0.173 | 0.155 | 0.345 | 0.047 | 0.654 | 0.703 | 1.000 | 0.130 | 1.141 | 0.282 | 163.9 | 7770.2 | 5979.4 | 630.6 | 4627.4 | 0.01 | 0.40 | 0.31 | 0.03 | 0.24 | 1.49 | 11.81 | 5.57 | 3.75 | 3.51 |
| **OSC18** | 0.190 | 0.169 | 0.350 | 0.030 | 0.683 | 0.736 | 1.000 | 0.144 | 1.151 | 0.293 | 141.3 | 8697.4 | 5943.3 | 729.5 | 5101.3 | 0.01 | 0.42 | 0.29 | 0.04 | 0.25 | 1.16 | 11.87 | 5.54 | 3.98 | 3.49 |
| **OSC19** | 0.201 | 0.204 | 0.377 | 0.031 | 0.700 | 0.754 | 1.000 | 0.151 | 1.170 | 0.307 | 107.2 | 8548.2 | 5839.8 | 789.1 | 4846.3 | 0.01 | 0.42 | 0.29 | 0.04 | 0.24 | 1.07 | 11.99 | 5.56 | 4.37 | 3.43 |
| **OSC20** | 0.233 | 0.236 | 0.384 | 0.041 | 0.732 | 0.770 | 1.000 | 0.187 | 1.191 | 0.331 | 136.5 | 8931.3 | 5952.5 | 1003.3 | 5041.7 | 0.01 | 0.42 | 0.28 | 0.05 | 0.24 | 1.21 | 12.04 | 5.59 | 5.11 | 3.44 |
| **OSC21** | 0.151 | 0.130 | 0.318 | 0.035 | 0.616 | 0.690 | 1.000 | 0.137 | 1.132 | 0.262 | 146.1 | 7416.6 | 5725.7 | 709.8 | 4633.6 | 0.01 | 0.40 | 0.31 | 0.04 | 0.25 | 1.13 | 11.49 | 5.67 | 4.55 | 3.48 |
| **OSC22** | 0.164 | 0.171 | 0.339 | 0.049 | 0.657 | 0.725 | 1.000 | 0.158 | 1.125 | 0.282 | 170.4 | 7641.3 | 5367.1 | 707.1 | 4445.5 | 0.01 | 0.42 | 0.29 | 0.04 | 0.24 | 1.51 | 11.76 | 5.56 | 4.16 | 3.42 |
| **OSC23** | 0.178 | 0.170 | 0.346 | 0.055 | 0.641 | 0.702 | 1.000 | 0.154 | 1.130 | 0.299 | 211.8 | 7225.5 | 5169.3 | 758.5 | 4188.5 | 0.01 | 0.41 | 0.29 | 0.04 | 0.24 | 1.63 | 11.81 | 5.53 | 4.64 | 3.55 |
| **OSC24** | 0.187 | 0.205 | 0.373 | 0.067 | 0.669 | 0.730 | 1.000 | 0.200 | 1.151 | 0.289 | 199.8 | 6511.1 | 4601.3 | 762.9 | 3860.4 | 0.01 | 0.41 | 0.29 | 0.05 | 0.24 | 1.86 | 12.01 | 5.61 | 4.04 | 3.39 |
| **OSC25** | 0.207 | 0.234 | 0.384 | 0.050 | 0.693 | 0.753 | 1.000 | 0.204 | 1.140 | 0.325 | 171.2 | 7848.0 | 5289.8 | 995.7 | 4368.3 | 0.01 | 0.42 | 0.28 | 0.05 | 0.23 | 1.43 | 11.93 | 5.44 | 4.85 | 3.46 |
| **OSC26** | 0.123 | 0.104 | 0.290 | 0.047 | 0.570 | 0.647 | 1.000 | 0.167 | 1.026 | 0.233 | 214.1 | 6178.1 | 4935.1 | 612.4 | 3773.8 | 0.01 | 0.39 | 0.31 | 0.04 | 0.24 | 1.44 | 11.64 | 5.49 | 3.59 | 3.56 |
| **OSC27** | 0.148 | 0.139 | 0.334 | 0.050 | 0.630 | 0.693 | 1.000 | 0.183 | 1.136 | 0.278 | 166.5 | 7604.3 | 5821.4 | 804.0 | 4638.0 | 0.01 | 0.40 | 0.31 | 0.04 | 0.24 | 1.64 | 11.64 | 5.60 | 4.12 | 3.47 |
| **OSC28** | 0.132 | 0.123 | 0.321 | 0.048 | 0.614 | 0.672 | 1.000 | 0.154 | 1.101 | 0.271 | 263.8 | 7352.7 | 5633.0 | 800.5 | 4556.3 | 0.01 | 0.39 | 0.30 | 0.04 | 0.25 | 1.77 | 11.73 | 5.48 | 4.30 | 3.51 |
| **OSC29** | 0.147 | 0.182 | 0.342 | 0.066 | 0.645 | 0.722 | 1.000 | 0.194 | 1.131 | 0.293 | 283.6 | 6915.6 | 5157.2 | 838.4 | 4214.6 | 0.02 | 0.40 | 0.30 | 0.05 | 0.24 | 1.75 | 11.55 | 5.48 | 4.01 | 3.44 |
| **OSC30** | 0.182 | 0.198 | 0.355 | 0.057 | 0.682 | 0.734 | 1.000 | 0.209 | 1.141 | 0.338 | 198.1 | 7393.4 | 5403.8 | 975.1 | 4346.1 | 0.01 | 0.40 | 0.30 | 0.05 | 0.24 | 1.71 | 11.86 | 5.56 | 4.38 | 3.43 |
| **OSC31** | 0.125 | 0.120 | 0.305 | 0.072 | 0.578 | 0.671 | 1.000 | 0.193 | 1.079 | 0.258 | 349.9 | 6684.6 | 5282.0 | 843.2 | 4117.2 | 0.02 | 0.39 | 0.31 | 0.05 | 0.24 | 2.32 | 11.42 | 5.55 | 4.32 | 3.52 |
| **OSC32** | 0.140 | 0.140 | 0.322 | 0.074 | 0.583 | 0.678 | 1.000 | 0.197 | 1.101 | 0.267 | 334.3 | 7041.8 | 5612.5 | 950.6 | 4251.8 | 0.02 | 0.39 | 0.31 | 0.05 | 0.23 | 2.28 | 11.41 | 5.50 | 4.30 | 3.40 |
| **OSC33** | 0.140 | 0.158 | 0.327 | 0.066 | 0.606 | 0.696 | 1.000 | 0.205 | 1.093 | 0.269 | 353.3 | 7781.2 | 5930.1 | 932.0 | 4738.4 | 0.02 | 0.39 | 0.30 | 0.05 | 0.24 | 1.96 | 11.39 | 5.45 | 4.11 | 3.50 |
| **OSC34** | 0.146 | 0.165 | 0.332 | 0.075 | 0.605 | 0.693 | 1.000 | 0.200 | 1.089 | 0.285 | 347.2 | 7347.0 | 5698.5 | 920.7 | 4458.0 | 0.02 | 0.39 | 0.30 | 0.05 | 0.24 | 2.22 | 11.36 | 5.40 | 4.62 | 3.46 |
| **OSC35** | 0.151 | 0.173 | 0.319 | 0.059 | 0.611 | 0.691 | 1.000 | 0.215 | 1.118 | 0.268 | 297.9 | 7625.8 | 5774.0 | 1162.8 | 4513.5 | 0.02 | 0.39 | 0.30 | 0.06 | 0.23 | 1.44 | 11.39 | 5.47 | 4.39 | 3.37 |
| **ORC1** | 0.186 | 0.144 | 0.377 | 0.027 | 0.691 | 0.699 | 1.000 | 0.082 | 1.181 | 0.278 | 28.4 | 4696.6 | 3666.0 | 301.9 | 3106.1 | 0.00 | 0.40 | 0.31 | 0.03 | 0.26 | 0.59 | 11.60 | 5.73 | 1.35 | 3.64 |
| **ORC2** | 0.270 | 0.350 | 0.416 | 0.138 | 0.752 | 0.823 | 1.000 | 0.404 | 1.164 | 0.412 | 375.6 | 6019.1 | 3739.0 | 1456.3 | 3296.4 | 0.03 | 0.40 | 0.25 | 0.10 | 0.22 | 2.25 | 11.99 | 5.38 | 4.89 | 3.47 |
| **ORC3** | 0.317 | 0.497 | 0.445 | 0.306 | 0.771 | 0.886 | 1.000 | 0.764 | 1.159 | 0.438 | 824.2 | 6139.9 | 3500.8 | 2666.0 | 3215.2 | 0.05 | 0.38 | 0.21 | 0.16 | 0.20 | 2.58 | 11.08 | 4.88 | 4.28 | 3.30 |
| **ORC4** | 0.455 | 0.793 | 0.577 | 0.506 | 0.867 | 1.034 | 1.000 | 1.231 | 1.128 | 0.601 | 1273.2 | 7080.4 | 3236.2 | 4080.7 | 2886.2 | 0.07 | 0.38 | 0.18 | 0.22 | 0.16 | 2.71 | 11.32 | 4.54 | 4.22 | 3.13 |
| **ORC5** | 0.580 | 1.061 | 0.730 | 0.701 | 0.948 | 1.315 | 1.000 | 1.635 | 1.109 | 0.734 | 1490.6 | 7257.9 | 2971.7 | 4978.4 | 2495.7 | 0.08 | 0.37 | 0.16 | 0.26 | 0.13 | 2.84 | 11.25 | 4.35 | 4.35 | 3.04 |
| **ORC6** | 0.160 | 0.112 | 0.352 | 0.026 | 0.661 | 0.692 | 1.000 | 0.088 | 1.161 | 0.266 | 31.3 | 5163.4 | 4047.8 | 331.2 | 3480.8 | 0.00 | 0.40 | 0.31 | 0.03 | 0.27 | 0.70 | 11.37 | 5.60 | 1.51 | 3.63 |
| **ORC7** | 0.259 | 0.282 | 0.384 | 0.165 | 0.730 | 0.786 | 1.000 | 0.459 | 1.133 | 0.372 | 396.6 | 5115.9 | 3391.5 | 1447.3 | 2911.2 | 0.03 | 0.39 | 0.26 | 0.11 | 0.22 | 2.60 | 11.63 | 5.58 | 4.61 | 3.58 |
| **ORC8** | 0.264 | 0.438 | 0.404 | 0.317 | 0.728 | 0.819 | 1.000 | 0.741 | 1.164 | 0.427 | 774.7 | 5112.4 | 3434.8 | 2546.4 | 2972.9 | 0.05 | 0.34 | 0.23 | 0.17 | 0.20 | 2.52 | 11.14 | 5.05 | 4.13 | 3.33 |
| **ORC9** | 0.380 | 0.665 | 0.533 | 0.465 | 0.810 | 0.959 | 1.000 | 1.078 | 1.146 | 0.529 | 1124.0 | 6215.1 | 3386.3 | 3576.1 | 2953.4 | 0.07 | 0.36 | 0.20 | 0.21 | 0.17 | 2.71 | 10.86 | 4.78 | 4.20 | 3.16 |
| **ORC10** | 0.533 | 0.990 | 0.660 | 0.674 | 0.907 | 1.101 | 1.000 | 1.648 | 1.087 | 0.698 | 1435.8 | 7077.2 | 3008.6 | 4832.8 | 2682.5 | 0.08 | 0.37 | 0.16 | 0.25 | 0.14 | 2.70 | 11.53 | 4.43 | 4.12 | 3.03 |
| **ORC11** | 0.148 | 0.097 | 0.326 | 0.030 | 0.626 | 0.683 | 1.000 | 0.095 | 1.109 | 0.244 | 27.1 | 4345.1 | 3615.9 | 315.2 | 3006.6 | 0.00 | 0.38 | 0.32 | 0.03 | 0.27 | 0.66 | 11.38 | 5.68 | 1.45 | 3.71 |
| **ORC12** | 0.227 | 0.288 | 0.367 | 0.162 | 0.670 | 0.760 | 1.000 | 0.399 | 1.085 | 0.352 | 424.9 | 5426.9 | 3630.0 | 1290.7 | 3070.8 | 0.03 | 0.39 | 0.26 | 0.09 | 0.22 | 2.43 | 11.84 | 5.44 | 4.42 | 3.55 |
| **ORC13** | 0.281 | 0.435 | 0.447 | 0.278 | 0.702 | 0.817 | 1.000 | 0.703 | 1.108 | 0.391 | 805.2 | 5629.1 | 3409.3 | 2419.5 | 2957.1 | 0.05 | 0.37 | 0.23 | 0.16 | 0.20 | 2.67 | 10.79 | 4.82 | 4.09 | 3.32 |
| **ORC14** | 0.342 | 0.684 | 0.476 | 0.425 | 0.796 | 0.942 | 1.000 | 1.142 | 1.070 | 0.528 | 1103.5 | 6164.8 | 3175.0 | 3690.5 | 2815.5 | 0.07 | 0.36 | 0.19 | 0.22 | 0.17 | 2.77 | 11.01 | 4.65 | 4.16 | 3.16 |
| **ORC15** | 0.457 | 0.919 | 0.616 | 0.616 | 0.903 | 1.119 | 1.000 | 1.527 | 1.050 | 0.680 | 1531.9 | 7493.4 | 3367.2 | 5147.3 | 2764.0 | 0.08 | 0.37 | 0.17 | 0.25 | 0.14 | 2.79 | 11.25 | 4.35 | 4.30 | 3.13 |
| **ORC16** | 0.145 | 0.088 | 0.319 | 0.047 | 0.607 | 0.685 | 1.000 | 0.112 | 1.108 | 0.275 | 155.4 | 5260.8 | 4182.9 | 383.6 | 3358.7 | 0.01 | 0.39 | 0.31 | 0.03 | 0.25 | 1.68 | 11.21 | 5.68 | 3.64 | 3.69 |
| **ORC17** | 0.231 | 0.306 | 0.370 | 0.149 | 0.690 | 0.766 | 1.000 | 0.427 | 1.064 | 0.358 | 427.0 | 5359.2 | 3515.6 | 1522.3 | 2856.5 | 0.03 | 0.39 | 0.26 | 0.11 | 0.21 | 2.43 | 11.71 | 5.30 | 4.79 | 3.37 |
| **ORC18** | 0.246 | 0.391 | 0.411 | 0.284 | 0.694 | 0.821 | 1.000 | 0.666 | 1.123 | 0.369 | 746.6 | 5583.7 | 3626.8 | 2223.9 | 2875.7 | 0.05 | 0.37 | 0.24 | 0.15 | 0.19 | 2.60 | 10.84 | 5.10 | 4.17 | 3.27 |
| **ORC19** | 0.347 | 0.598 | 0.504 | 0.419 | 0.752 | 0.942 | 1.000 | 1.007 | 1.104 | 0.492 | 1053.7 | 6200.2 | 3389.0 | 3335.2 | 2947.2 | 0.06 | 0.37 | 0.20 | 0.20 | 0.17 | 2.66 | 11.10 | 4.68 | 4.12 | 3.15 |
| **ORC20** | 0.500 | 0.910 | 0.632 | 0.635 | 0.914 | 1.148 | 1.000 | 1.493 | 1.090 | 0.673 | 1320.1 | 6871.1 | 2998.0 | 4609.0 | 2560.8 | 0.07 | 0.37 | 0.16 | 0.25 | 0.14 | 2.71 | 11.20 | 4.50 | 4.42 | 2.95 |
| **ORC21** | 0.117 | 0.100 | 0.313 | 0.056 | 0.589 | 0.665 | 1.000 | 0.141 | 1.134 | 0.205 | 142.0 | 4875.2 | 4016.4 | 435.3 | 3242.0 | 0.01 | 0.38 | 0.32 | 0.03 | 0.26 | 1.85 | 11.60 | 5.67 | 3.88 | 3.56 |
| **ORC22** | 0.177 | 0.250 | 0.352 | 0.179 | 0.649 | 0.755 | 1.000 | 0.405 | 1.103 | 0.326 | 530.2 | 5629.1 | 3950.2 | 1437.5 | 3204.6 | 0.04 | 0.38 | 0.27 | 0.10 | 0.22 | 2.81 | 11.35 | 5.47 | 4.69 | 3.42 |
| **ORC23** | 0.229 | 0.372 | 0.375 | 0.305 | 0.671 | 0.816 | 1.000 | 0.654 | 1.127 | 0.351 | 909.0 | 5984.5 | 3808.5 | 2330.6 | 3112.0 | 0.06 | 0.37 | 0.24 | 0.14 | 0.19 | 2.81 | 10.77 | 5.05 | 4.07 | 3.25 |
| **ORC24** | 0.301 | 0.581 | 0.473 | 0.484 | 0.738 | 0.938 | 1.000 | 0.991 | 1.077 | 0.482 | 1202.3 | 6644.2 | 3407.7 | 3379.9 | 2818.2 | 0.07 | 0.38 | 0.20 | 0.19 | 0.16 | 2.94 | 11.03 | 4.74 | 4.34 | 3.20 |
| **ORC25** | 0.427 | 0.831 | 0.558 | 0.625 | 0.869 | 1.091 | 1.000 | 1.394 | 1.069 | 0.656 | 1422.7 | 7501.0 | 3395.0 | 4527.5 | 2723.8 | 0.07 | 0.38 | 0.17 | 0.23 | 0.14 | 2.87 | 11.11 | 4.51 | 4.27 | 2.94 |
| **ORC26** | 0.142 | 0.110 | 0.304 | 0.079 | 0.576 | 0.656 | 1.000 | 0.200 | 1.049 | 0.246 | 241.2 | 5102.3 | 4159.9 | 630.7 | 3231.6 | 0.02 | 0.38 | 0.31 | 0.05 | 0.24 | 2.50 | 11.01 | 5.50 | 4.16 | 3.52 |
| **ORC27** | 0.197 | 0.252 | 0.342 | 0.187 | 0.607 | 0.711 | 1.000 | 0.451 | 1.045 | 0.300 | 526.8 | 5152.1 | 3698.5 | 1485.1 | 3050.2 | 0.04 | 0.37 | 0.27 | 0.11 | 0.22 | 2.58 | 11.29 | 5.38 | 4.58 | 3.37 |
| **ORC28** | 0.235 | 0.396 | 0.394 | 0.277 | 0.668 | 0.795 | 1.000 | 0.633 | 1.070 | 0.384 | 834.2 | 5897.6 | 3654.0 | 2273.2 | 2952.4 | 0.05 | 0.38 | 0.23 | 0.15 | 0.19 | 2.62 | 11.23 | 4.95 | 4.24 | 3.16 |
| **ORC29** | 0.283 | 0.535 | 0.455 | 0.441 | 0.701 | 0.884 | 1.000 | 0.934 | 1.040 | 0.440 | 1163.2 | 6045.9 | 3587.8 | 3183.7 | 2965.7 | 0.07 | 0.36 | 0.21 | 0.19 | 0.18 | 2.87 | 10.50 | 4.76 | 4.23 | 3.27 |
| **ORC30** | 0.429 | 0.835 | 0.577 | 0.574 | 0.823 | 1.030 | 1.000 | 1.308 | 1.050 | 0.617 | 1367.6 | 6847.8 | 3272.7 | 4425.9 | 2577.1 | 0.07 | 0.37 | 0.18 | 0.24 | 0.14 | 2.90 | 11.05 | 4.54 | 4.34 | 2.86 |
| **ORC31** | 0.124 | 0.110 | 0.313 | 0.094 | 0.568 | 0.671 | 1.000 | 0.207 | 1.034 | 0.271 | 236.2 | 4072.6 | 3298.5 | 484.1 | 2564.5 | 0.02 | 0.38 | 0.31 | 0.05 | 0.24 | 2.04 | 11.17 | 5.46 | 4.14 | 3.55 |
| **ORC32** | 0.166 | 0.248 | 0.328 | 0.201 | 0.611 | 0.742 | 1.000 | 0.479 | 1.098 | 0.295 | 622.9 | 5439.2 | 3892.6 | 1604.4 | 3011.6 | 0.04 | 0.37 | 0.27 | 0.11 | 0.21 | 2.66 | 10.89 | 5.31 | 3.78 | 3.26 |
| **ORC33** | 0.218 | 0.420 | 0.391 | 0.312 | 0.646 | 0.808 | 1.000 | 0.686 | 1.033 | 0.366 | 808.9 | 5472.7 | 3435.8 | 2061.3 | 2721.8 | 0.06 | 0.38 | 0.24 | 0.14 | 0.19 | 2.67 | 10.94 | 5.02 | 3.95 | 3.25 |
| **ORC34** | 0.299 | 0.525 | 0.467 | 0.416 | 0.727 | 0.894 | 1.000 | 0.930 | 1.092 | 0.461 | 990.2 | 5969.7 | 3465.9 | 2973.4 | 2740.5 | 0.06 | 0.37 | 0.22 | 0.18 | 0.17 | 2.77 | 10.84 | 4.89 | 4.18 | 3.20 |
| **ORC35** | 0.442 | 0.833 | 0.605 | 0.546 | 0.826 | 1.070 | 1.000 | 1.346 | 1.061 | 0.639 | 1356.7 | 7140.6 | 3563.1 | 4654.0 | 2721.8 | 0.07 | 0.37 | 0.18 | 0.24 | 0.14 | 2.77 | 10.98 | 4.46 | 4.14 | 2.91 |
